# A benchmark study of deep learning-based multi-omics data fusion methods for cancer

**DOI:** 10.1186/s13059-022-02739-2

**Published:** 2022-08-09

**Authors:** Dongjin Leng, Linyi Zheng, Yuqi Wen, Yunhao Zhang, Lianlian Wu, Jing Wang, Meihong Wang, Zhongnan Zhang, Song He, Xiaochen Bo

**Affiliations:** 1 Institute of Health Service and Transfusion Medicine, Beijing, People’s Republic of China; 2 School of Informatics, Xiamen University, Xiamen, People’s Republic of China; 3 Academy of Medical Engineering and Translational Medicine, Tianjin University, Tianjin, People’s Republic of China; 4 School of Medicine, Tsinghua University, Beijing, People’s Republic of China

## Abstract

**Background:**

A fused method using a combination of multi-omics data enables a comprehensive study of complex biological processes and highlights the interrelationship of relevant biomolecules and their functions. Driven by high-throughput sequencing technologies, several promising deep learning methods have been proposed for fusing multi-omics data generated from a large number of samples.

**Results:**

In this study, 16 representative deep learning methods are comprehensively evaluated on simulated, single-cell, and cancer multi-omics datasets. For each of the datasets, two tasks are designed: classification and clustering. The classification performance is evaluated by using three benchmarking metrics including accuracy, F1 macro, and F1 weighted. Meanwhile, the clustering performance is evaluated by using four benchmarking metrics including the Jaccard index (JI), C-index, silhouette score, and Davies Bouldin score. For the cancer multi-omics datasets, the methods’ strength in capturing the association of multi-omics dimensionality reduction results with survival and clinical annotations is further evaluated. The benchmarking results indicate that moGAT achieves the best classification performance. Meanwhile, efmmdVAE, efVAE, and lfmmdVAE show the most promising performance across all complementary contexts in clustering tasks.

**Conclusions:**

Our benchmarking results not only provide a reference for biomedical researchers to choose appropriate deep learning-based multi-omics data fusion methods, but also suggest the future directions for the development of more effective multi-omics data fusion methods. The deep learning frameworks are available at https://github.com/zhenglinyi/DL-mo.

**Supplementary Information:**

The online version contains supplementary material available at 10.1186/s13059-022-02739-2.

## Background

Advances in high-throughput techniques have led to an explosion of multi-omics data in biomedical research. Each type of omics data helps researchers to understand the complex biological systems from different perspectives, such as genomics, transcriptomics, proteomics, and metabolomics [1]. Researchers have utilized omics data to address key biomedical problems, such as personalized complex disease therapy [2, 3], drug discovery [4, 5], and cancer drug target discovery [6, 7]. Multi-omics data allow researchers to comprehensively understand biologic systems from different aspects because these omics have complementary roles and work together to perform a certain biological function. However, multi-omics data are complex, high-dimensional, and heterogeneous [8, 9], and it is challenging to extract valuable knowledge from these multi-omics data. To address this challenge, various methods have been developed, such as multiple kernel learning, Bayesian consensus clustering, machine learning (ML)-based dimensionality reduction, similarity network fusion, and deep learning (DL) methods [10, 11].

Some researchers reviewed and tested several traditional ML algorithms from a data fusion perspective [10, 12–15]. Rappoport et al. [10] evaluated the methods including multiple kernel learning, Bayesian consensus clustering, ML-based dimension reduction, and similarity network fusion. Tini et al. [15] and Pierre-Jean et al. [14] evaluated the methods including Bayesian consensus clustering, ML-based dimension reduction, and similarity network fusion. Cantini et al. tested and discussed nine joint dimensionality reduction methods [12]. Chauvel et al. focused on the Bayesian consensus clustering and dimension reduction methods [13]. According to the above evaluations, each of these ML methods performs differently on different datasets and tasks. As a rapidly developing branch in the field of ML, DL utilizes efficient algorithms to process complex, high-dimensional, and heterogeneous data. Compared to traditional ML algorithms, DL can better capture nonlinearities and complex relationships in multi-omics data. However, few benchmark studies comprehensively compare the performance of various DL methods.

This paper evaluated the performance of 16 representative and open-source models from all DL-based data fusion methods on three different datasets, i.e., simulated multi-omics datasets, single-cell multi-omics datasets, and cancer multi-omics datasets. These 16 models were grouped into two categories: supervised models (six) and unsupervised models (ten). Accordingly, for each of the datasets, two tasks were designed: classification and clustering. For simulated and single-cell datasets, ground-truth samples were retrieved through classification and clustering by using six supervised models and ten unsupervised models, respectively. For cancer datasets, supervised DL methods were evaluated in the classification tasks on five types of cancer datasets with ground-truth cancer subtypes. Meanwhile, unsupervised DL methods were evaluated in the clustering tasks. Furthermore, the associations of the embeddings with survival and clinical annotations were evaluated. Based on the benchmarking results, we provided recommendations for biologists to choose appropriate methods in different scenarios and give guidelines on methodological improvements for researches focusing on algorithm design of multi-omics data fusion.

## Results

### DL-based multi-omics data fusion methods and benchmarking workflow

**Fig. 1:**
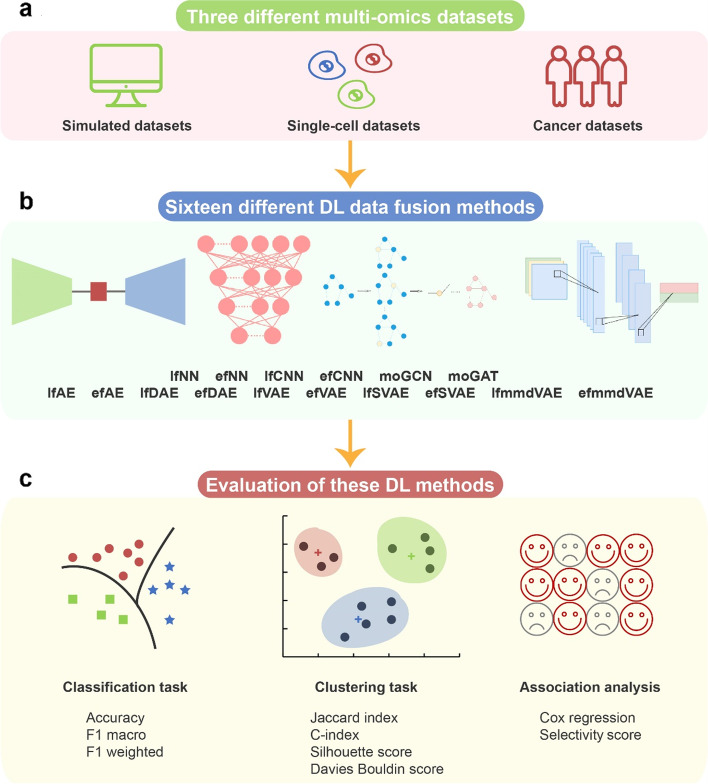
Schematic of the benchmarking workflow. **a** Three different multi-omics datasets cover simulated, single-cell, and cancer multi-omics datasets. **b** 16 DL methods were used to fuse the multi-omics data. **c** The DL methods were evaluated in various scenarios

DL-based multi-omics data fusion methods aim to learn low-dimensional embeddings from the fusion of multi-omics data for various downstream tasks. According to our investigation, various DL-based data fusion methods can achieve this goal, including fully connected neural network (FCNN) [16–20], convolutional neural network (CNN) [21–23], autoencoder (AE) [24–34], graph neural network (GNN) [35–39], capsule network (CapsNet) [40, 41], generative adversarial network (GAN) [42], and mixture DL-based models for multi-omics data fusion [43, 44]. Most of these models in previous publications were used with different strategies (early or late fusion). Early fusion means each omics data are fused first and then inputted into DL-based models. Late fusion means the multi-omics data are inputted into DL-based models first and then fused for downstream tasks. Because of the difference in input omics data and downstream tasks, it is difficult to compare these methods directly. To make different methods comparable, in this study, we first extracted the data fusion part from the original model and then compared the performance on unified datasets and tasks. We selected multi-omics data fusion methods according to the following two rules: (1) The original models of the selected methods have open-source code, and (2) the original models used multi-omics fusion and the data fusion part of the original model can be extracted separately so that we can evaluate the data fusion on unified datasets and tasks. To comprehensively evaluate the performance of the models, three types of multi-omics datasets were used in this study: simulated data, single-cell data, and cancer data. Notably, the evaluation models can be grouped into two categories: supervised models and unsupervised models. Therefore, for each of the datasets, two tasks were designed: classification for supervised models and clustering for unsupervised models. Classification performance was evaluated using three benchmarking metrics, namely accuracy, F1 macro, and F1 weighted. Clustering performance was evaluated using four benchmarking metrics, namely Jaccard index (JI), C-index, silhouette score, and Davies Bouldin score. Furthermore, for the cancer multi-omics datasets, this paper further evaluated the methods’ ability to capture the association of multi-omics dimensionality reduction results with survival and clinical annotations. The associations could reflect representational ability and interpretability of the fused low-dimensional embeddings (Fig. 1, Table 1).

**Table 1:** Evaluation of the DL-based multi-omics data fusion methods and metrics

Dataset		Evaluation model	Task type	Evaluation metrics
**Simulated multi-omics datasets**		lfNN, efNN, lfCNN, efCNN, moGCN, moGAT	Classification	1. Accuracy 2. F1 macro 3. F1 weighted
		lfAE, efAE, lfDAE, efDAE, lfVAE, efVAE, lfSVAE, efSVAE, lfmmdVAE, efmmdVAE	Clustering	1. Jaccard Index 2. C-index 3. Silhouette score 4. Davies Bouldin score
**Single-cell multi-omics datasets**		lfNN, efNN, lfCNN, efCNN, moGCN, moGAT	Classification	1. Accuracy 2. F1 macro 3. F1 weighted
		lfAE, efAE, lfDAE, efDAE, lfVAE, efVAE, lfSVAE, efSVAE, lfmmdVAE, efmmdVAE	Clustering	1. Jaccard Index 2. C-index 3. Silhouette score 4. Davies Bouldin Score
**Cancer multi-omics datasets**	BRCA, GBM, SARC, LUAD, STAD	lfNN, efNN, lfCNN, efCNN, moGCN, moGAT	Classification	1. Accuracy 2. F1 macro 3. F1 weighted
	AML, BRCA, COAD, GBM, KIRC, LIHC, LUSC, SKCM, OV, SARC	lfAE, efAE, lfDAE, efDAE, lfVAE, efVAE, lfSVAE, efSVAE, lfmmdVAE, efmmdVAE	Clustering	1. C-index 2. Silhouette score 3. Davies Bouldin Score
			Association of embeddings with survival	Cox proportional-hazards regression
			Association of embeddings with clinical annotations	Selectivity score

*BRCA* breast cancer, *GBM* glioblastoma, *SARC* sarcoma, *LUAD* lung adenocarcinoma, *STAD* stomach cancer, *AML* acute myeloid leukemia, *BRCA* breast cancer, *COAD* colon cancer, *GBM* glioblastoma, *KIRC* kidney clear cell carcinoma, *LIHC* kidney chromophobe, *LUSC* lung squamous cell carcinoma, *SKCM* melanoma, *OV* ovarian cancer, *SARC* sarcoma

To make the structural differences of different models easy to understand, we renamed the model according to the structural characteristics (early or late fusion). The evaluation models are described as follows: a late fusion method based on AutoEncoder (lfAE), an early fusion method based on AutoEncoder (efAE), a late fusion method based on Denoising AutoEncoder (lfDAE), an early fusion method based on Denoising AutoEncoder (efDAE), an early fusion method based on Variational AutoEncoder (efVAE), an early fusion method based on Stacked Variational AutoEncoder (efSVAE), an efVAE method whose loss function is a maximum mean discrepancy (efmmdVAE), a late fusion method based on Neural Network (lfNN), an early fusion method based on Neural Network (efNN), a late fusion method based on Convolutional Neural Network (lfCNN), an early fusion method based on Convolutional Neural Network (efCNN), a multi-omics Graph Convolutional Network method (moGCN), and a multi-omics Graph Attention network method (moGAT). To accommodate different evaluation data, this study modified the input of these ten original models proposed by Ma et al. [24], Lee et al. [26], Poirion et al. [28], Guo et al. [29], Zhang et al. [33], Ronen et al. [32], Hira et al. [34], Kuru et al. [20], Preuer et al. [19], Islam et al. [22], Fu et al. [21], Wang et al. [38], and Xing et al. [39], respectively. Specifically, Ma et al. [24] used the late fusion AEs on gene expression, miRNA expression, and DNA methylation data to develop a robust model to predict clinical target variables. Lee et al. [26] developed an early fusion AE model and fused gene expression, miRNA expression, DNA methylation, and CNV data to predict lung adenocarcinoma survival rate. Poirion et al. [28] used gene expression, miRNA expression, and DNA methylation as input to predict the survival subtypes in bladder cancer by utilizing the late fusion DAE algorithm. Guo et al. [29] fed gene expression, miRNA expression, and CNV data into a novel framework to robustly identify ovarian cancer subtypes using the early fusion DAEs. Zhang et al. [33] used the early fusion VAE to classify samples from DNA methylation and gene expression profiles. Ronen et al. [32] developed an early fusion SVAE and used gene expression, miRNA expression, and DNA methylation to classify cancer subtypes. Hira et al. [34] used gene expression, miRNA expression, and DNA methylation as input and improved the loss function of early fusion VAE to analyze ovarian cancer through patient stratification analysis. The models proposed by Kuru et al. [20], Preuer et al. [19], Islam et al. [22], Fu et al. [21], Wang et al. [38], and Xing et al. [39] are supervised. Kuru et al. [20] and Preuer et al. [19] used gene expression and the drug pairs’ chemical structure data as input to predict drug synergy. Their models are based on the late and early fusion FCNN, respectively. Islam et al. developed a late fusion CNN and used CNV, gene expression, and clinical data to classify molecular subtypes. Fu et al. used variation counts, gene expression, QTANs/QTALs number, and WGCNA module features as the input of an early fusion CNN to predict gene regulation mechanisms. Wang et al. [38] introduced a novel multi-omics data fusion method for biomedical classification and used gene expression, DNA methylation, miRNA expression data, and the corresponding similarity network as input to train a GCN to generate initial predictions for the category labels. Xing et al. [39] used a gene co-expression network as the input of GAT for disease diagnosis and prognosis.

Furthermore, considering evaluation completeness, our own frameworks were designed, including a late fusion method based on Variational Autoencoder (lfVAE), a late fusion method based on Stacked Variational Autoencoder (lfSVAE), and a lfVAE method with a loss function of maximum mean discrepancy (lfmmdVAE).

### Evaluation of DL-based multi-omics data fusion methods on simulated datasets

**Fig. 2:**
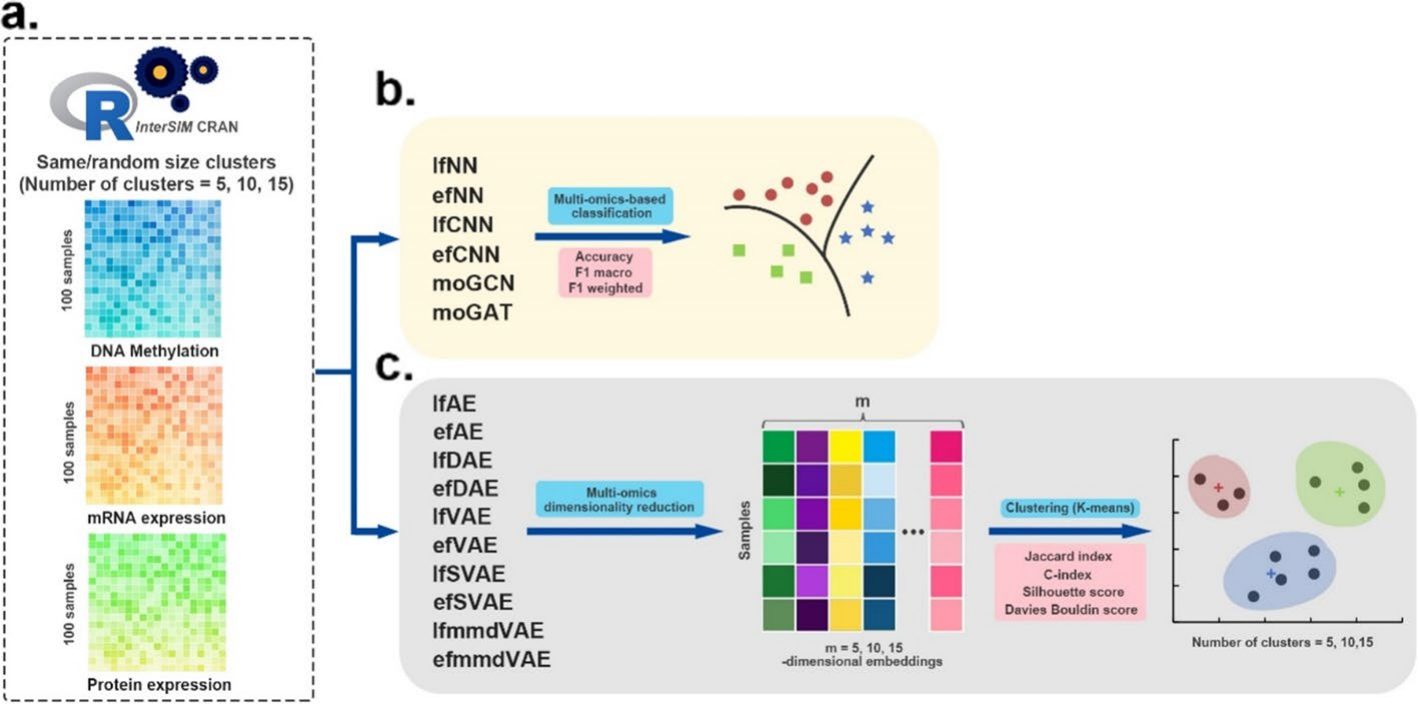
Workflow of the evaluation on simulated multi-omics datasets. **a** *InterSIM* CRAN package generated three kinds of omics data that were used as input. **b** Supervised DL methods are evaluated in the classification tasks. The performance of these methods was based on 4-fold cross-validation and was evaluated by three metrics: accuracy, F1 macro, and F1 weighted score. **c** Unsupervised DL methods are applied to fuse the simulated multi-omics data to obtain 5-dimensional, 10-dimensional, and 15-dimensional embeddings first. Then k-means algorithm is used to cluster the multi-omics dimensionality reduction results. We employed JI, C-index, silhouette score and Davies Bouldin score as the evaluation indexes of clustering

This study first evaluated DL-based multi-omics fusion methods on simulated multi-omics datasets that were generated using the *InterSIM* CRAN package [45] (Fig. 2). This package can generate complex and interrelated multi-omics data, including DNA methylation, mRNA gene expression, and protein expression data. One hundred simulated samples with 1000-dimensional features were generated. In generation process, the cluster number parameter of 100 simulated samples is set to 5, 10, and 15. Furthermore, we generated each cluster of samples in two conditions: all clusters have the same size, or the clusters have variable random sizes. This simulates a real application scenario in which the proportion of samples belonging to each cluster (subtype) could be the same or different.

Six supervised DL methods are evaluated in the classification tasks. These six supervised methods are intrinsically designed for sample classification, and they classify the samples of ground-truth clusters (subtypes). Their performances were then compared based on the classification results. For the ten other unsupervised DL methods, they were applied to fuse the simulated multi-omics data to obtain 5-dimensional, 10-dimensional, and 15-dimensional embeddings first. The dimension of the embeddings was set according to the number of clusters in the simulated multi-omics data. Then, the *k*-means algorithm was adopted to cluster the multi-omics dimensionality reduction results. The clustering of the samples was finally obtained to compare the performances of the ten unsupervised methods.

To quantitatively evaluate the performances of the six supervised DL methods, we partitioned the dataset into training and test sets at the ratio of 3:1 and performed 4-fold cross-validation. Meanwhile, three metrics (accuracy, F1 macro, and F1 weighted score) were calculated (see “Methods”). It can be seen that the efNN, moGCN, and moGAT achieved better classification performance with higher accuracy, F1 macro, and F1 weighted values (Table 2, Additional file 1: Table S1). The performance of the two GNN-based methods (moGCN and moGAT) is remarkable. The two CNN-based methods (efCNN and lfCNN) are less effective on this benchmark. This indicates that using CNN with a one-dimensional convolution layer on the input vector may not be optimal in multi-omics data fusion.

**Table 2:** Performance of six supervised methods in the condition that all clusters have the same size

Methods	5 clusters of the same size			10 clusters of the same size			15 clusters of the same size		
	Accuracy	F1 macro	F1 weighted	Accuracy	F1 macro	F1 weighted	Accuracy	F1 macro	F1 weighted
lfNN	**1.0**	**1.0**	**1.0**	0.900	0.860	0.875	0.860	0.792	0.818
efNN	**1.0**	**1.0**	**1.0**	**1.0**	**1.0**	**1.0**	**1.0**	**1.0**	**1.0**
lfCNN	**1.0**	**1.0**	**1.0**	0.760	0.707	0.696	0.880	0.751	0.835
efCNN	**1.0**	**1.0**	**1.0**	**1.0**	**1.0**	**1.0**	0.920	0.911	0.893
moGCN	**1.0**	**1.0**	**1.0**	**1.0**	**1.0**	**1.0**	**1.0**	**1.0**	**1.0**
moGAT	**1.0**	**1.0**	**1.0**	**1.0**	**1.0**	**1.0**	**1.0**	**1.0**	**1.0**

**Fig. 3:**
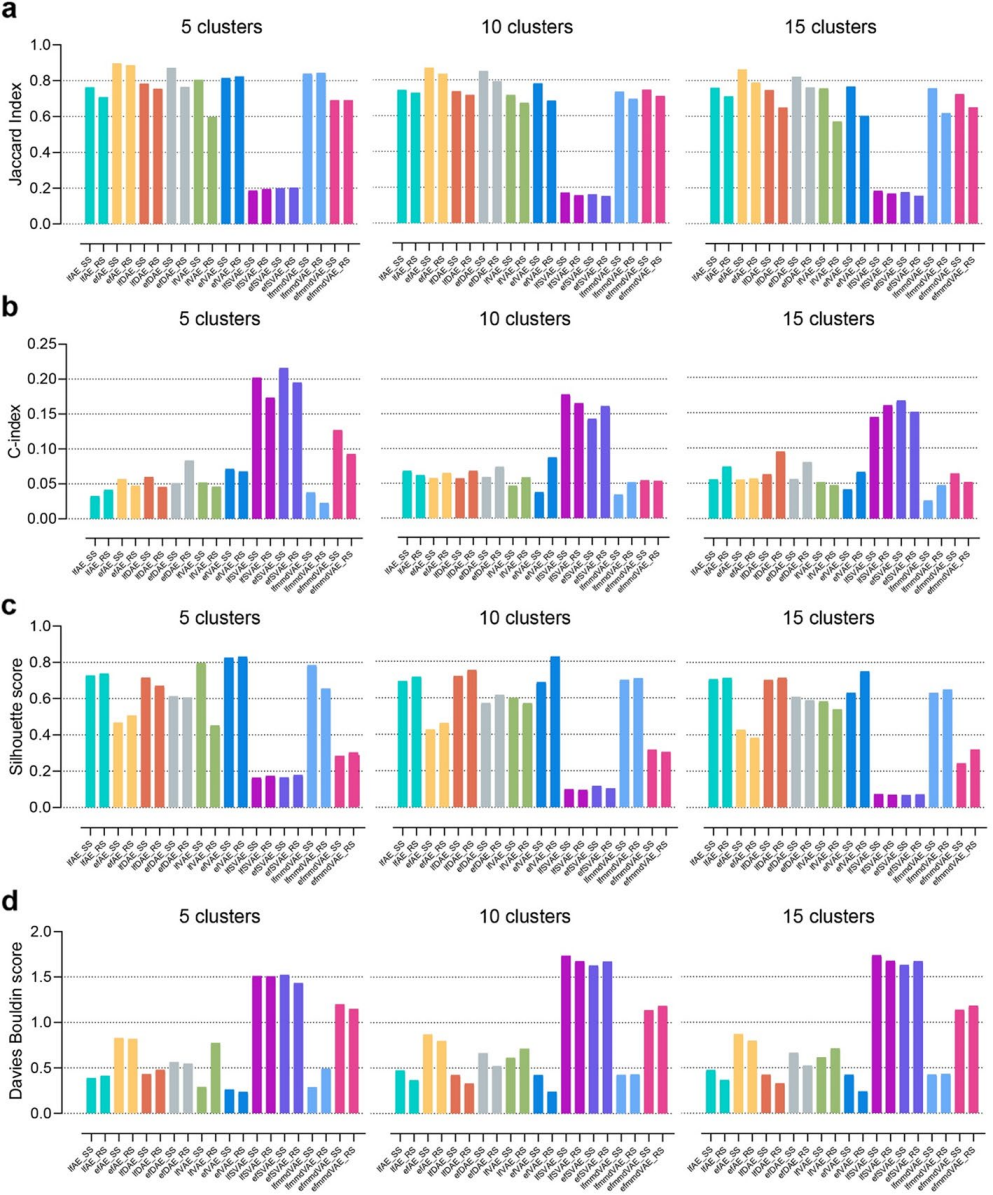
JI, C-index, silhouette score, and Davies Bouldin score of the ten unsupervised methods for clustering on simulated multi-omics datasets. An external index JI (**a**) and three internal indices C-index, silhouette score and Davies Bouldin score (**b**, **c**, **d**) were calculated based on the clustering on the simulated data. The cluster number is set to 5, 10, and 15. SS and RS represent two conditions, i.e., all clusters have the same size and the clusters have variable random sizes. The k-means clustering was run over 1000 times. The results of **a** are presented as mean values of JIs

For the clustering tasks, JI was employed to measure the consistency between multi-omics data fusion-based clusters and the ground-truth clusters. JI is an external comparison index used to measure the similarity and diversity of sample sets. The value of the JI ranges from 0 to 1, and the higher the value, the better the clustering result. It can be seen from the experimental results that most methods showed stable performance on different numbers of clusters (Fig. 3a, Additional file 1: Table S2). Compared with the condition that clusters have variable random sizes, most methods obtained higher JI values in the condition that all clusters have the same size. Most of the methods performed reasonably well in different simulated scenarios (JIs >0.6), except for the SVAE methods. According to JI, efAE, efDAE, and efVAE are overall the best-performing methods. These methods are among the top three best methods in 6/6, 5/6, and 3/6 simulated scenarios, according to JI.

In addition to the external comparison index (JI), this paper also employed three internal indices (C-index, silhouette score, and Davies Bouldin score) to evaluate the clustering performance (Fig. 3b,c,d, Additional file 1: Table S3,S4,S5). The internal indices were used to measure the goodness of a clustering structure without external information [46]. The values of the C-index, silhouette score, and Davies Bouldin score range from 0 to 1, −1 to 1, and 0 to infinity. The lower the C-index and Davies Bouldin score are, the better the clustering results are. While the higher silhouette score indicates better clustering result. According to C-index, lfmmdVAE achieves the best performance and is among the top three methods in 6/6 scenarios. According to the silhouette score and Davies Bouldin score, efVAE is the best-performing method. Meanwhile, the two SVAE methods obtain the worst performance according to the three internal indices.

### Evaluation of DL-based multi-omics data fusion methods on single-cell datasets

Applying multi-omics data fusion methods to single-cell multi-omics data helps to systematically explore the heterogeneity of cells [47]. To further benchmark the performances of DL-based multi-omics data fusion methods, it is crucial to evaluate these methods on single-cell multi-omics data.

**Fig. 4:**
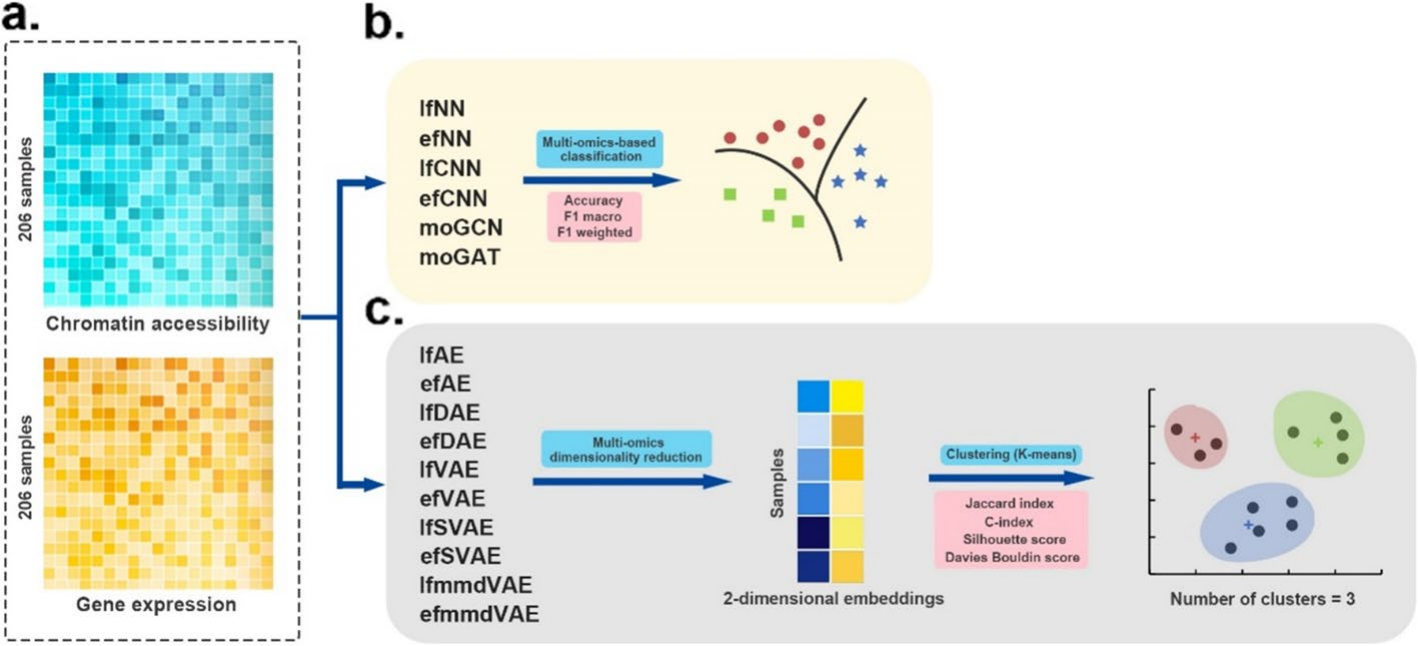
Workflow of the evaluation on single-cell multi-omics datasets. **a** Two kinds of omics data were used as input. **b** Supervised DL methods are evaluated in the classification tasks. The performance of these methods was based on 4-fold cross-validation and was evaluated by three metrics: accuracy, F1 macro, and F1 weighted score. **c** Unsupervised DL methods were first applied to fuse the single-cell multi-omics data to obtain the fused two-dimensional embeddings. Then k-means algorithm was used to cluster the multi-omics dimensionality reduction results into three categories. We employed JI, C-index, silhouette score and Davies Bouldin score as the evaluation indexes of clustering

The single-cell datasets consist of two omics data types, i.e., single-cell chromatin accessibility data and single-cell gene expression data (Fig. 4). The number of features for these two types of omics data is 49,073 and 207,203, respectively. And the two omics data were obtained from three different cancer cell lines (HTC, Hela, and K562) for a total of 206 cells [48].

Similar to the evaluation on the simulated multi-omics data above, this study first evaluated six supervised classification methods on the single-cell dataset. These methods classified the samples of three cancer cell lines. The performance of these methods was obtained based on 4-fold cross-validation and was evaluated by three metrics: accuracy, F1 macro, and F1 weighted score (Table 3). It can be seen that the results are similar to those on the simulated data sets. lfNN, efNN, moGCN, and moGAT all perform very well.

**Table 3:** Performance of six supervised methods on single-cell multi-omics datasets

	Accuracy	F1 macro	F1 weighted
lfNN	**1.0**	**1.0**	**1.0**
efNN	**1.0**	**1.0**	**1.0**
lfCNN	0.962	0.952	0.962
efCNN	0.981	0.976	0.981
moGCN	**1.0**	**1.0**	**1.0**
moGAT	**1.0**	**1.0**	**1.0**

**Fig. 5:**
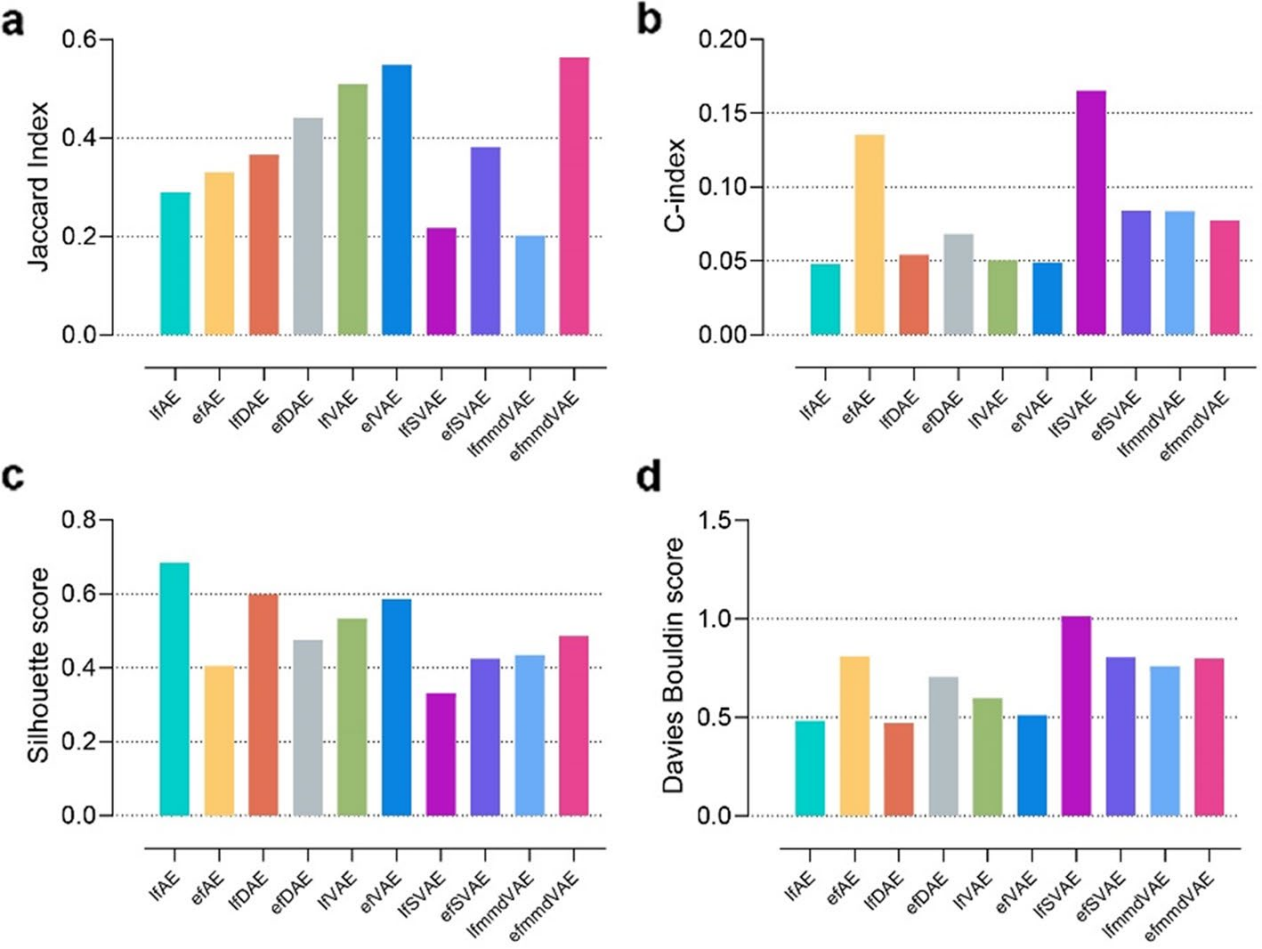
JI, C-index, silhouette score, and Davies Bouldin score of the ten unsupervised methods for clustering on single-cell multi-omics datasets. An external index JI (**a**) and three internal indices C-index, silhouette score and Davies Bouldin score (**b**, **c**, **d**) were calculated based on the clustering on the single-cell data. The cluster number is three. The k-means clustering was run over 1000 times. The results of **a** are presented as mean values of JIs

For the clustering tasks, ten unsupervised DL methods were first applied to fuse the single-cell multi-omics data to obtain the fused two-dimensional embeddings. Then, the *k*-means algorithm was employed to cluster the multi-omics dimensionality reduction results into three categories. The clustering of the samples was finally obtained to compare the performances of the ten unsupervised methods. This study adopted JI, C-index, silhouette score, and Davies Bouldin score as the evaluation indexes of clustering. According to the external index JI, efmmdVAE and efVAE are the best-performing methods (Fig. 5, Additional file 1: Table S6). According to the three internal indices, lfAE, lfDAE, and efmmdVAE achieve good performance. Overall, efmmdVAE and lfAE are among the top three methods in 3/4 evaluation indices, so they are the most promising methods on this benchmark.

### Evaluation of DL-based multi-omics data fusion methods on cancer datasets

In recent years, the rapid development of high-throughput sequencing technologies enables researchers to obtain multi-omics molecular profiles of various cancer types. To better understand the molecular and clinical characteristics of cancers, it is crucial to use multi-omics data fusion methods [49].

**Fig. 6:**
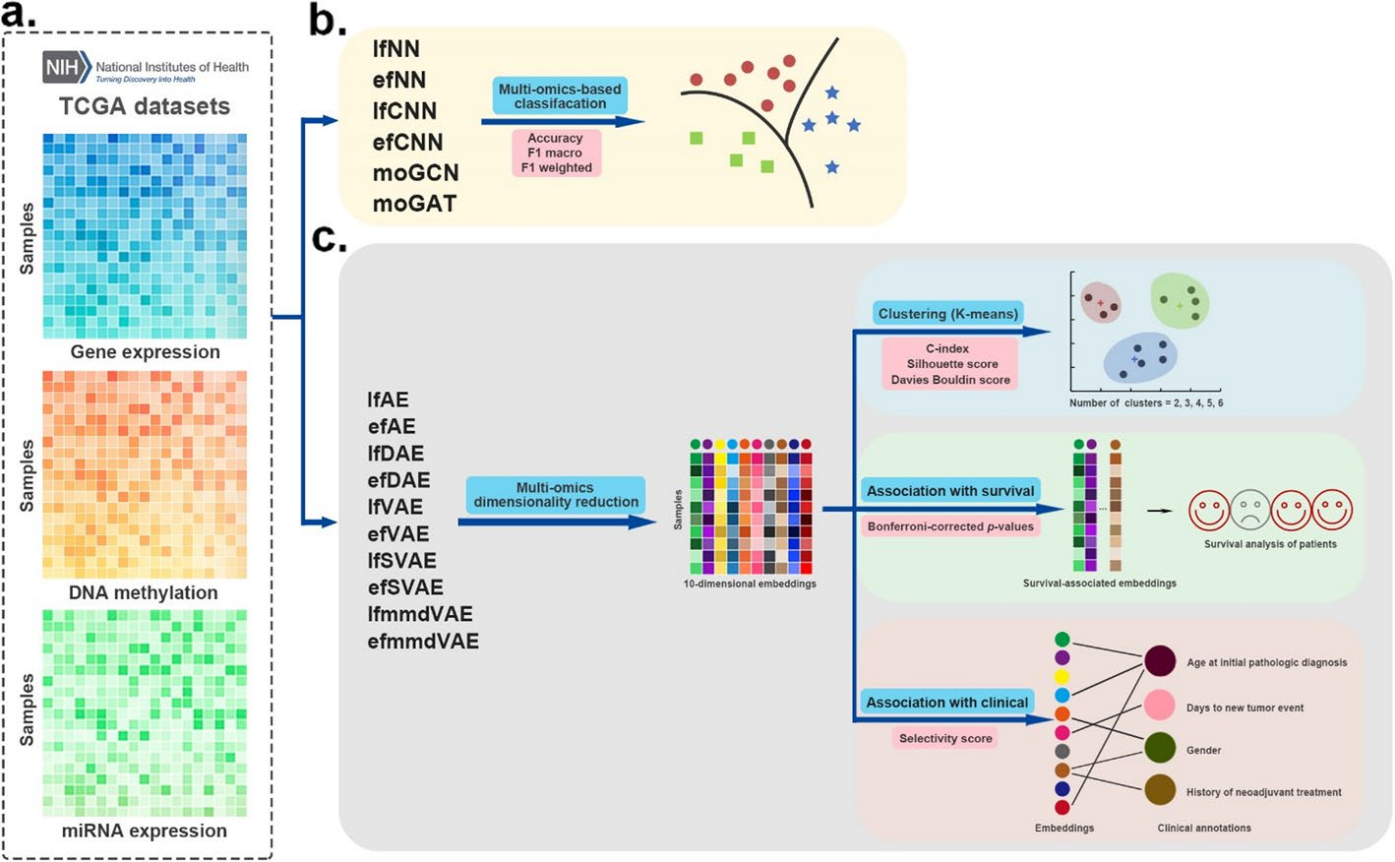
Workflow of the evaluation on cancer multi-omics datasets. **a** Three kinds of omics data were used as input. **b** Supervised DL methods are evaluated in the classification tasks. The performance of these methods was based on 4-fold cross-validation and was evaluated by three metrics: accuracy, F1 macro, and F1 weighted score. **c** Unsupervised DL methods were first applied to fuse the cancer multi-omics data to obtain the fused 10-dimensional embeddings. Then k-means algorithm was used to cluster the multi-omics dimensionality reduction results into several categories. We employed C-index, silhouette score and Davies Bouldin score as the evaluation indexes of clustering. Furthermore, the associations of the embeddings with survival and clinical annotations were evaluated

This study evaluated DL-based multi-omics fusion methods on The Cancer Genome Atlas (TCGA) cancer multi-omics datasets (Fig. 6). The datasets consist of three omics data types: gene expression, DNA methylation, and miRNA expression. For the classification tasks, we collected five different cancer datasets with ground-truth cancer subtypes from TCGA, including breast cancer (BRCA), glioblastoma (GBM), sarcoma (SARC), lung adenocarcinoma (LUAD), and stomach cancer (STAD). For the clustering task, to ensure the authenticity of the evaluation, the data used in this study were obtained from benchmark cancer datasets (http://acgt.cs.tau.ac.il/multi_omic_benchmark/download.html) [10].

Similar to the evaluation on the simulated multi-omics data and single-cell multi-omics data above, six supervised classification methods were firstly evaluated on the five different cancer datasets with ground-truth cancer subtypes. These methods classified the samples of different ground-truth cancer subtypes (Table 4). The performance of these methods was obtained based on 4-fold cross-validation and was evaluated by three metrics: accuracy, F1 macro, and F1 weighted score (see “Methods”). For each cancer data set, the samples with all three omics data types were selected, and 59, 272, 206, 144, and 198 samples were obtained for BRCA, GBM, SARC, LUAD, and STAD, respectively. The subtypes for each cancer are listed in Table 4. Among the five supervised methods (Table 5), moGAT obtain the most promising results on BRCA and GBM. moGCN, lfNN, and efNN achieve the best performance on SARC, LUAD, and STAD, respectively. lfCNN obtains the lowest scores of all the three metrics on 3/5 datasets. In this benchmark, the GNN-based methods show great advantages.

**Table 4:** The sizes and features of cancer benchmark datasets used in classification task

Cancers	Categories (cancer subtypes)	# of samples	# of features
			Exp, Meth, miRNA
BRCA	Luminal A: 28, Luminal B: 15, Basal-like: 12, HER2-enriched: 4	59	6000, 5000, 892
GBM	Proneural: 71, Classical: 70, Mesenchymal: 84, Neural: 47	272	6000, 5000, 534
SARC	DDLPS: 50, LMS: 80, UPS: 44, MFS: 17, MPNST: 5, SS: 10	206	6000, 5000, 1046
LUAD	TRU: 51, PI: 52, PP: 41	144	6000, 5000, 554
STAD	EBV: 20, MSI: 38, GS: 43, CIN: 97	198	6000, 5000, 519

# number, *Exp* gene expression, *Meth* DNA methylation, *miRNA* miRNA expression, *DDLPS* dedifferentiated liposarcoma, *LMS* leiomyosarcoma, *UPS* undifferentiated pleomorphic sarcoma, *MFS* myxofibrosarcoma, *MPNST* malignant peripheral nerve sheath tumor, *SS* synovial sarcoma, *TRU* formerly bronchioid, *PI* formerly squamoid, *PP* formerly magnoid, *EBV* Epstein–Barr virus, *MSI* microsatellite instability, *GS* genomically stable, *CIN* chromosomal instability

**Table 5:** Performance of six supervised methods on cancer benchmark datasets used in classification task

Methods	BRCA			GBM			SARC			LUAD			STAD		
	Accuracy	F1 macro	F1 weighted	Accuracy	F1 macro	F1 weighted	Accuracy	F1 macro	F1 weighted	Accuracy	F1 macro	F1 weighted	Accuracy	F1 macro	F1 weighted
lfNN	0.475	0.161	0.306	0.309	0.118	0.146	0.767	0.544	0.739	**0.896**	**0.892**	**0.894**	0.843	0.853	0.844
efNN	0.660	0.544	0.617	0.588	0.512	0.554	0.772	0.668	0.771	0.861	0.848	0.855	**0.868**	**0.876**	**0.866**
lfCNN	0.525	0.248	0.382	0.548	0.435	0.481	0.587	0.328	0.525	0.688	0.683	0.683	0.657	0.543	0.606
efCNN	0.594	0.431	0.546	0.455	0.347	0.372	0.786	0.626	0.768	0.868	0.866	0.868	0.818	0.834	0.818
moGCN	0.600	**0.589**	0.601	0.647	0.633	0.585	**0.846**	**0.852**	**0.851**	0.861	0.860	0.861	0.740	0.733	0.716
moGAT	**0.667**	0.545	**0.671**	**0.721**	**0.702**	**0.73**	0.731	0.719	0.735	0.861	0.861	0.863	0.820	0.779	0.819

In addition, it can be seen that all methods do not perform well on BRCA. This is because BRCA has significantly fewer samples than other cancers. For GBM, although it has the largest number of samples among the five cancers, most methods do not achieve good performance. According to investigation, it is found that the subtype labels of GBM may have some deviations. Recent studies suggest that GBM should be classified into three subtypes instead of the four subtypes labeled by TCGA [50–53].

To further explore how the data size influences the benchmarks, we reduced the amount of data and observed the effects of data reduction. Specifically, 20%, 40%, 60%, and 80% of the total samples in the original data were randomly selected. Then, all six methods were evaluated under different amounts of data. The results of the data reduction experiment are illustrated in Fig. S1. Except for two GNN-based methods (moGAT and moGCN), the performances of other methods are impaired when the amount of data decreases. The performance of the two GNN-based methods fluctuates greatly. This may be because the network structure changes greatly with the change of data for the GNN-based methods.

**Fig. 7:**
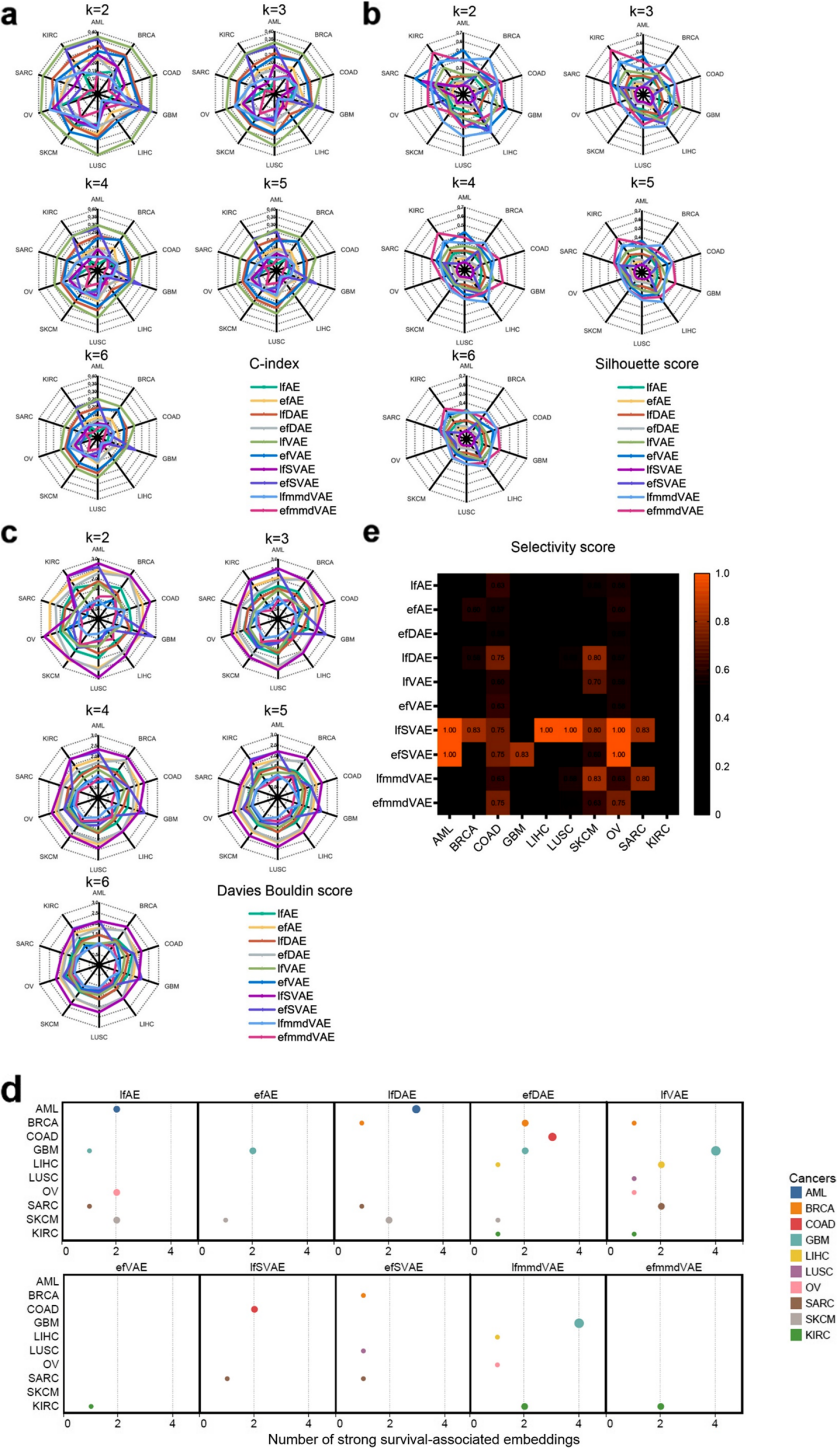
C-index, silhouette score, Davies Bouldin score, and the association of the embeddings with survival and clinical annotations for the ten unsupervised methods on cancer multi-omics datasets. C-index (**a**), silhouette score (**b**) and Davies Bouldin score (**c**) were calculated based on the clustering on the cancer data. The number of clusters is set from two to six. The k-means clustering was run over 1000 times. **d** The embeddings which had strong association with survival (the Bonferroni-corrected p-values smaller than 0.05). The X-axis represents the number of survival-associated embeddings. The Y-axis represents cancers, and every cancer is assigned a color. **e** Selectivity score of the ten unsupervised methods for ten different cancer types. The score is displayed if it is higher than the average score (0.49), and the higher the selectivity score, the brighter the orange block

For the clustering tasks, ten unsupervised DL methods were first applied to fuse the cancer multi-omics data to obtain the fused 10-dimensional embeddings. The embedding dimension was set as that in the work of Bismeijer et al. [54] and Cantini et al. [12]. Then, the *k*-means algorithm was employed to cluster the multi-omics dimensionality reduction results into several categories. Because the optimal cluster number (the ground-truth cancer subtypes) was uncertain, the number of clusters was set from two to six in this study. Finally, the clustering of the samples was obtained to compare the performances of the ten unsupervised methods. This study adopted the C-index, silhouette score, and Davies Bouldin score as the evaluation indexes of clustering (Fig. 7a, b, c, Additional file 1: Table S7, S8, S9). Note that JI was not used as the evaluation index because of the lack of information on ground-truth cancer subtypes in the benchmark cancer datasets. Among these ten DL methods, efmmdVAE, efVAE, and lfmmdVAE are the best-performing methods. They are among the top three best methods in 42/50, 41/50, and 21/50 datasets according to C-index, in 47/50, 42/50, and 43/50 according to silhouette score, and in 46/50, 39/50, and 48/50 according to Davies Bouldin score, respectively. In particular, efmmdVAE outperforms the other methods in terms of C-index, silhouette score, and Davies Bouldin score on KIRC.

**Fig. 8:**
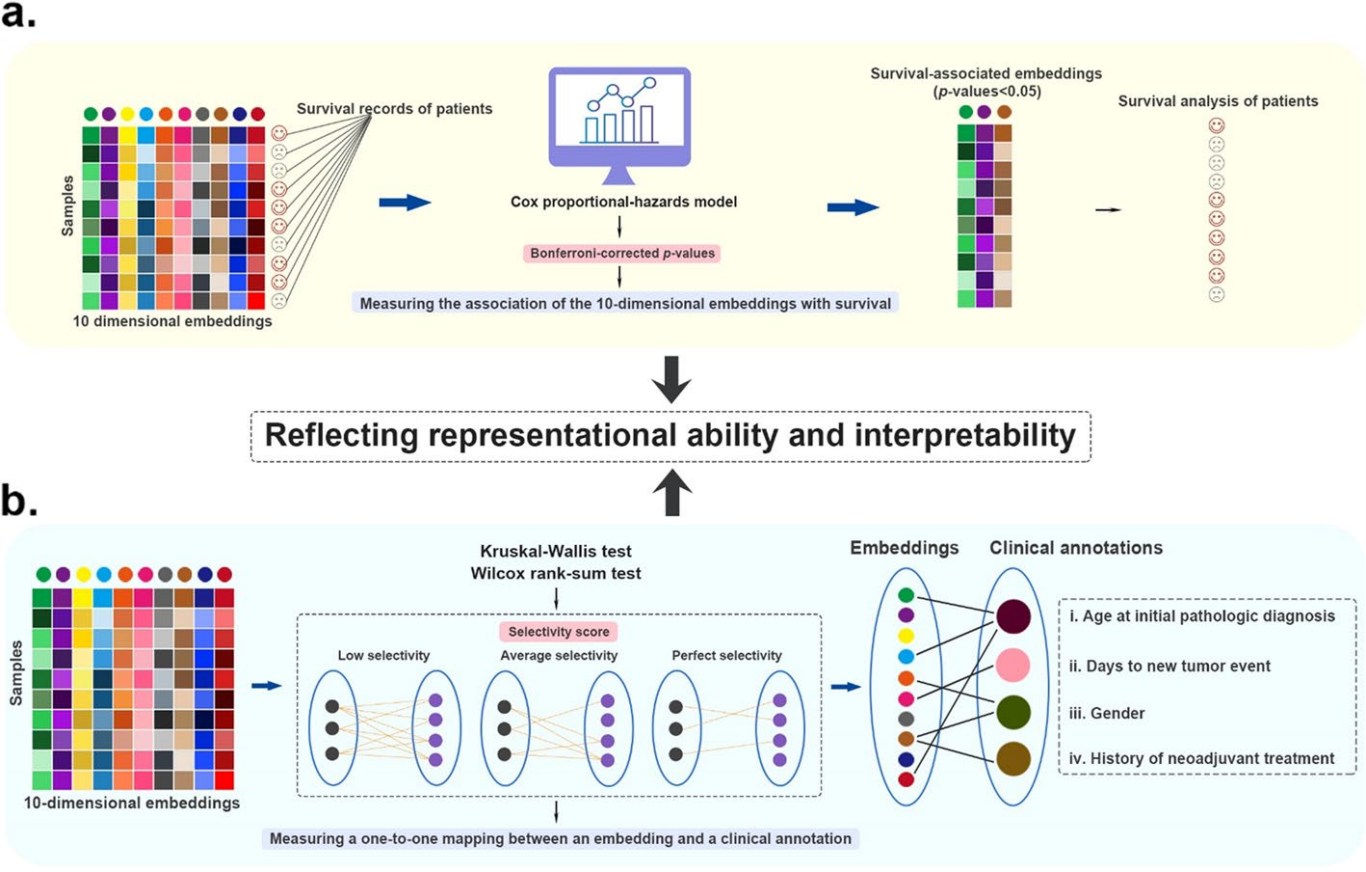
Graphic summary of the cancer sub-benchmark. **a** The details of testing the association of embeddings with survival. **b** The details of testing the association of embeddings with clinical annotations

To further evaluate the effect of data fusion by using these DL-based methods, we not only used the fused ten-dimensional embeddings for clustering analysis but also evaluated the associations of the embeddings with survival and clinical annotations (Fig. 8). On the one hand, the associations could reflect representational ability of the fused ten-dimensional embeddings, and on the other hand, the associations partly reflect the interpretability of the embeddings.

To evaluate the association of the embeddings with survival, we employed the Cox proportional-hazards regression model and calculated Bonferroni-corrected *p*-values. The Bonferroni-corrected *p*-values indicate to what extent an embedding can distinguish the difference between the population survival conditions. The statistical significance threshold was set to 0.05. The embeddings with strong association with survival (the Bonferroni-corrected *p*-values smaller than 0.05) are illustrated in Fig. 7d. The more this type of embeddings, the better the performance of the method. In fact, survival is a comprehensive clinical characteristic affected by many factors. For example, the survival markers of poor prognosis for adrenocortical cancer can be various types of genes, miRNA, and DNA methylation signatures [55–58]. Similar to the markers, the embeddings with strong association with survival can reflect their impact on survival from various aspects.

Based on the results, we observed that the number of embeddings associated with survival depended on not only the DL method but also the cancer type. In three cancer types (GBM, KIRC, and SARC), half of the DL methods identify at least one survival-associated embedding. In general, lfVAE and efDAE achieve the best performance, and they can find embeddings significantly associated with survival in 7/10 and 6/10 cancer types, respectively.

Subsequently, using the same ten-dimensional embeddings described above, we evaluated the association of the embeddings with clinical annotations (Fig. 7e, Additional file 1: Table S10). Four clinical annotations were selected, i.e., “age at initial pathologic diagnosis,” “days to new tumor event after initial treatment,” “gender,” and “history of neoadjuvant treatment.” The Kruskal-Wallis test and Wilcoxon rank-sum were adopted to test the significance of the associations of the embeddings with these clinical annotations. Specifically, the Kruskal-Wallis test was used for “age at initial pathologic diagnosis” and “days to new tumor event after initial treatment,” and the Wilcoxon rank-sum was used for “gender” and “history of neoadjuvant treatment.” Different from the association of the embeddings with survival mentioned above, the association of the embeddings with clinical annotations is expected to be a one-to-one mapping, i.e., an embedding is associated with a clinical annotation. In this way, each embedding is interpretable. Therefore, after obtaining the strong associations through Kruskal-Wallis test and Wilcoxon rank-sum, we employed the selectivity score as the evaluation metric [12]. The selectivity score falls within [0, 1]. When each embedding is associated with one and only one clinical annotation, the selectivity score is 1. When all embeddings are associated with all clinical annotations, the selectivity score is 0. The top methods are those with selectivity scores above the average. The results indicate that the average selectivity score of all methods across all cancer types is 0.49. The selectivity scores of all DL methods for kidney cancer are 0. In particular, the selectivity score of lfSVAE is 1 for AML LIHC, LUSC, and OV; the selectivity score of efSVAE is 1 for AML and OV. Overall, lfSVAE, efSVAE, lfAE, lfDAE, efAE, and lfmmdVAE are among the top methods for 8/10, 6/10, 6/10, 6/10, 6/10, and 6/10 cancer types, respectively. The six methods are the best-performing methods on these ten cancer datasets. Although the two SVAE methods do not perform well in clustering performance, they have an advantage in finding the meaningful embeddings that are associated with clinical annotations.

## Discussion and conclusions

Increasing evidence has shown that multi-omics data analysis plays an important role in a wide spectrum of biomedical research, which has promoted the development of multi-omics data fusion methods. Here, this study systematically evaluated 16 DL-based methods that are representative multi-omics fusion methods in three different contexts, i.e., simulated multi-omics datasets, single-cell multi-omics datasets, and cancer multi-omics datasets. For each of the datasets, two tasks were designed: classification and clustering. Meanwhile, various evaluation metrics were employed to evaluate the models’ performance from different aspects.

When evaluated on simulated multi-omics datasets, most supervised methods show good performances in classification tasks, especially efNN, moGCN, and moGAT. The two CNN-based methods (efCNN and lfCNN) are less effective on this benchmark, indicating that using CNN with a one-dimensional convolution layer on the input vector may not be suitable for multi-omics data fusion. For the clustering task, efAE, lfmmdVAE, and efVAE show the best performance. Similar to the result on simulated datasets, moGCN and moGAT perform very well on the classification task of single-cell datasets. As for the evaluation of the clustering performance on the single-cell dataset, efmmdVAE and lfAE are the most efficient methods. Finally, on the cancer data benchmark, moGAT still outperforms the other supervised methods on the classification task. When evaluating the clustering performance, efmmdVAE, efVAE, and lfmmdVAE achieve the most promising results in most scenarios. When evaluating the associations of the embeddings with survival or clinical annotations, lfVAE and lfSVAE are the most efficient. Therefore, for the study of embedding-level information, lfVAE and lfSVAE are worthy of being prioritized.

**Fig. 9:**
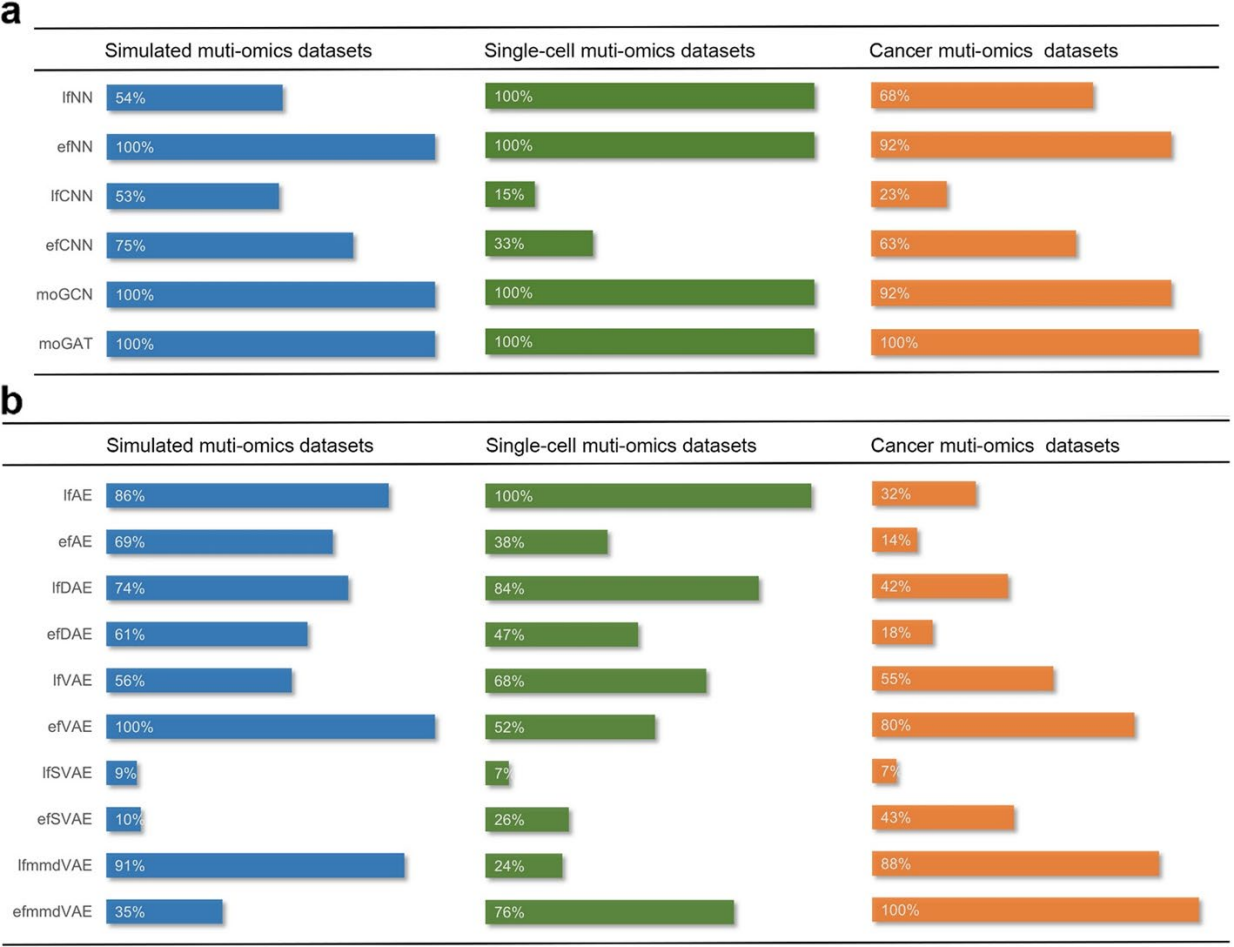
DL-based multi-omics data fusion methods benchmarked by average unified score in this study. **a** The unified performances of supervised models in three different datasets. **b** The unified performances of unsupervised models in three different datasets. We used the highest unified score of every scenario as reference (marked 100%) to calculate the percentage

Based on the above results, to make our evaluation more objective, we defined a unified score (see “Methods”) and ranked these DL methods according to the unified score. If one method was evaluated in more than one scenario, its average unified scores were used. For the classification tasks, moGAT ranks first on the three different multi-omics datasets (Fig. 9). For the clustering tasks, efVAE, lfmmdVAE, and lfAE are the top three methods on the simulated datasets. lfAE, lfDAE, and efmmdVAE are the top three methods on single-cell datasets. efmmdVAE, lfmmdVAE, and efVAE are the top three methods on cancer datasets. Overall, GNN-based methods should be prioritized by researchers focusing on classification tasks. GNN-based methods structure the multi-omics data into similarity networks. The correlations among samples can be captured by the similarity networks. Therefore, the omics features and the geometrical structures of the data can be effectively utilized and the classification performance can benefit from it. When focusing on the clustering tasks, efmmdVAE, efVAE, and lfmmdVAE should be prioritized. They have the most effective and consistent behaviors across all the different benchmarks. These methods learn the probability distribution of the data. They have a layer of data means and standard deviations, which are used to generate new data. This allows for better generalization and flexibility of the learned embeddings. They can thereby be valuable tools for researchers who are interested in applying DL-based multi-omics data fusion methods to various biomedical problems.

Although the state-of-the-art DL-based multi-omics data fusion methods are evaluated comprehensively on three different datasets by employing various evaluation indices and scenarios, there is still limitation in this benchmark study. Combining all omics types may introduce noise because there may be information redundancy in different omics data. The compatibility of omics data should be checked to avoid the case that different omics data is completely discordant. In the future, different combinations of omics data and the selection of a less-redundant set of omics data will be considered in this benchmark study.

Despite the great progress in multi-omics data fusion brought by the above DL methods, there is still room for future improvement from a computational perspective. (1) Dealing with class imbalance. As demonstrated in the evaluation on the simulated datasets, most methods perform better in the condition that all clusters have the same size than in the condition that clusters have variable random sizes. Imbalanced classes could impair model performance. Further extensions of DL-based multi-omics data fusion methods could handle class imbalance problems by applying cost-sensitive learning [59, 60], ensemble learning (e.g., bagging and boosting), etc. (2) Combining AE and GNN. The evaluation results indicate that GNN-based methods achieve good performance. Graph autoencoder (GAE), which combines AE and GNN, has achieved success on many tasks [61–63]. Applying GAE-based methods to cancer and single-cell multi-omics data fusion could be promising and is worthy of further exploration. (3) Designing algorithms that accommodate the missing observations. Multi-omics data fusion is often accompanied by the absence of samples in one or several omics. Taking more omics types into consideration and using the sample sets consisting of all omics data types can lead to a limited sample size. One solution to this problem is to infer the missing features. Based on the observation that different omics are not completely independent and can be correlated, the missing features can be inferred by using the complementary information of different omics. For example, in the field of chemoinformatics, Martino et al. designed a model by employing a siamese neural network to massively infer the missing features for ~800,000 molecules [64]. In addition to missing feature inference, using generative adversarial networks (GAN) to generate data similar to a real dataset is also a promising method. Since GAN-based algorithms can learn and imitate any distribution of data, Ahmed et al. [42] employed GAN to fuse two omics data, but this GAN-based model can only be applied to specific types of data with explicit interactions (e.g., miRNA-mRNA interaction). Although GAN-based multi-omics data fusion algorithms have some limitations currently, they deserve to be further explored in the application of missing value imputation [65]. (4) Developing explainable DL methods. Most of the state-of-the-art DL methods lack interpretation, which is increasingly demanded in the biomedical field. For the “black-box” of DL models, it is difficult to elucidate the underlying biological mechanisms. One emerging approach is to embed prior biological knowledge into the DL models. Several studies used knowledge-embedded algorithms to provide explanations [66–68]. For example, Mao et al. [66] performed dimensionality reduction on high-dimensional single-cell transcriptome data and provided explanations by embedding prior knowledge into matrix factorization. Gut et al. [67] proposed a knowledge-embedded VAE by restricting the structure of VAE to mirror gene-pathway memberships and applied the model to reduce the dimensionality of single-cell RNA-seq data. Similarly, developing knowledge-embedded DL methods for multi-omics data fusion is promising and can provide new insight into the underlying mechanisms. Based on this comprehensive benchmark study and several potential improvement strategies, more progress can be achieved on multi-omics data fusion.

## Methods

### Presentation of the ten evaluation models

This study considered *p* omics matrices *X*_*i*_ (*i* = 1, …, *p*) with a dimension of *m* × *n*_*i*_ (*m* samples and *n*_*i*_ features). Each sample can be represented by *p* vectors *x*_*i*_ (*i* = 1, …, *p*). Note that the original models of all methods in this part can be found in the publications [19–22, 24, 26, 28, 29, 32–34, 38, 39].

### FCNN

FCNN is a simulated neural network and usually consists of an input layer, multiple hidden layers, and an output layer. The neurons in the hidden layers receive the multi-dimensional input vector *X* and output *y*, which can be expressed in Eq. (1).

1 $${y}^1=\sigma \left({W}^1X+{b}^1\right)$$

where *y*^1^ is the first output vector, *W*^1^ and *b*^1^ are parameters that can be learned according to the input *X*, *σ* is the activation function. The multilayer neural network can be expressed in Eq. (2).

2 $${y}^l=\sigma \left({W}^l{y}^{l-1}+{b}^l\right)$$

This study used two types of FCNN with different structures, i.e., efNN and lfNN.

*efNN:* The *p*-omics vectors are concatenated into one feature vector *X*. The dimension of *X* is ${\sum}_{i=1}^P{n}_i$.

3 $$X= concat\left({x}_1,{x}_2,\dots {x}_p\right)$$

The vector *X* is used as the input of a multilayer neural network for classification; *relu* is used for the activation function in the middle layers, and *softmax* is used in the last layer. *relu* and *softmax* are expressed in Eq. (4) and Eq. (5), respectively.

4 $$relu(z)=\mathit{\operatorname{Max}}\left(0,z\right)$$

5 $$softmax=\frac{e^{a_i}}{\sum_{j=1}^n{e}^{a_j}}$$

where *n* is the number of features.

Therefore, the middle and last layers can be expressed in Eq. (6) and Eq. (7), respectively.

6 $${y}^l= relu\left({W}^l{y}^{l-1}+{b}^l\right)$$

7 $${y}^{out}= softmax\left({W}^{out}{y}^{out-1}+{b}^{out}\right)$$

The overall loss function is the cross-entropy loss *L*_*ce*_, which can be expressed in Eq. (8).

8 $${L}_{ce}=-\frac{1}{m}{\sum}_{i=1}^m{\sum}_{j=1}^l{y}_j^{(i)}\log {\hat{y}}_j^{(i)}$$

where *m* is the number of samples, and *l* is the number of categories.

*lfNN*: Each omics vector *x*_*i*_ is used as the input to a subnetwork. The outputs *o*_*i*_ of the subnetwork are the intermediate features of each type of omics data. Then, the outputs of multiple neural networks are concatenated into a vector *O*.

9 $$O= concat\left({o}_1,{o}_2,\dots, {o}_p\right)$$

Then, the vector *O* is used as the input of a multilayer neural network for classification. The evaluation is consistent with that of *efNN*, except that the last layer uses the *softmax* activation function, and the other layers use *relu*. The loss function is also a cross-entropy loss *L*_*ce*_.

### CNN

CNN stimulates the biomimetic biological natural cognition mechanism, and it is a neural network learning framework using image visual computing. A typical CNN mainly consists of convolutional layers, activation layers, pooling layers, and fully connected layers. The convolution layer is mainly composed of multiple convolution kernels. The convolution kernel is to operate the kernel function on the local image, and its essence is the discrete convolution between two two-dimensional matrices. The operation principle is as follows:

10 $$\mathrm{Cov}\left(x,y\right)={\textstyle\sum_{t=0}^k}{\textstyle\sum_{s=0}^k}F\left(s,t\right)\times G\left(x-s,y-t\right).$$

where *s* and *t* are the widths of the convolution kernel in the *x* and *y* directions, *F* is the parameter matrix of the convolution kernel, *G* is the local image matrix that is operated with the convolution kernel, and *k* is the size of the convolution kernel.

The pooling layer is to reduce data dimensionality, thereby decreasing the number of parameters and calculation amount inside the CNN. Meanwhile, it can prevent the network from overfitting to a certain extent. Usually, the maximum pooling layer is used in CNN, that is, the maximum value in the local receptive field is taken. Its mathematical description is as follows:

11 $$P=\left\{\underset{w\times w}{\max }{A}^{l\times l}\right\}$$

where *P* is the feature matrix obtained by max pooling, *l* is the width of the feature map, *A* is the feature matrix after activation of the convolutional layer, and *w* is the width of the pooling region.

The One-dimensional Convolutional Neural Network (1DCNN) is essentially the same as convolutional neural networks. Although 1DCNN has only one dimension, it also has the advantages of translation invariance of CNN for feature recognition. Structurally, 1DCNN is almost the same as CNN. It also includes a series of convolutional layers and pooling layers and outputs the results through the fully connected layer. The difference is that in the calculation of convolutional layers and pooling layers, 1DCNN only extracts the feature sequence of the one-dimensional sequence. The operations of the convolutional layer and the pooling layer in the 1DCNN are as follows:

12 $$\mathrm{Cov}\left(x,y\right)={\textstyle\sum_{a=0}^w}F(a)\times G\left(x-a\right)$$

13 $$P=\left\{\underset{w}{\max }{A}^l\right\}$$

This study used two types of CNN with different structures, namely efCNN and lfCNN.

*efCNN:* It is similar to efNN, and this study added convolutional layers and pooling layers to the network structure. The p-omics vectors are concatenated into one feature vector *X*. After the convolution layer and the pooling layer, the output features are flattened into and out of the fully connected network to make the final prediction.

*lfCNN:* It is similar to lfNN. Each omics vector *x*_*i*_ is used as the input to a subnetwork. Each subnetwork consists of convolutional layers and pooling layers. The outputs of each subnetwork are flattened, concatenated, and fed into a fully connected neural network to make the final prediction.

### moGCN

GCNs are used for omics-specific learning in moGCN, and a GCN is trained for each type of omics data to perform classification tasks. A GCN model takes two inputs. One input is a feature matrix *X* ∈ *ℝ*^*n* × *d*^, where *n* is the number of nodes, and *d* is the number of input features. The other input is a description of the graph structure, which can be represented as an adjacency matrix *A* ∈ *ℝ*^*n* × *n*^. A GCN can be constructed by stacking multiple convolutional layers. Specifically, each layer is defined as:

14 $${H}^{\left(l+1\right)}=f\left({H}^{(l)},A\right)=\sigma \left(A{H}^{(l)}{W}^{(l)}\right)$$

where *H*^(*l*)^ is the input of the *l*th layer, *W*^(*l*)^ is the weight matrix of the *l*th layer, and *σ*(·) denotes a non-linear activation function. To train GCNs effectively, the adjacency matrix *A* is further modified as:

15 $$\overset{\sim }{A}={\hat{D}}^{-\frac{1}{2}}\hat{A}{\hat{D}}^{-\frac{1}{2}}={\hat{D}}^{-\frac{1}{2}}\left(A+I\right){\hat{D}}^{-\frac{1}{2}}$$

where $\hat{D}$ is the diagonal node degree matrix of $\hat{A}$, and *I* is the identity matrix.

The original adjacency matrix *A* is obtained by calculating the cosine similarity between pairs of nodes, and edges with cosine similarity larger than a threshold *ϵ* are retained. Specifically, the adjacency between node *i* and node *j* in the graph is calculated as:

16 $$A_{ij}=\left\{\begin{array}{l}s\left(x_i,x_j\right),\kern0.5em if\;i\neq j\;and\;s\left(x_i,x_j\right)\geq\epsilon\\0,\kern0.5em otherwise\end{array}\right.$$

where *x*_*i*_ and *x*_*j*_ are the feature vectors of nodes *i* and *j*, respectively. $s\left({x}_i,{x}_j\right)=\frac{x_i{x}_j}{\parallel {x}_i{\parallel}_2\parallel {x}_j{\parallel}_2}$ is the cosine similarity between nodes *i* and *j*.

To perform omics-specific classification, a multilayer GCN is constructed for each omics data type. Specifically, for the *i*th omics data type, an omics-specific GCN, i.e., *GCN*_*i*_(·), is trained with training data ${X}_{tr}^{(i)}\in {\mathbb{R}}^{n_{tr}\times {d}_i}$ and the corresponding adjacency matrix ${\overset{\sim }{A}}_{tr}^{(i)}\in {\mathbb{R}}^{n_{tr}\times {n}_{tr}}$. The predictions on the training data can be expressed as:

17 $${\hat{Y}}_{tr}^{(i)}= GC{N}_i\left({X}_{tr}^{(i)},{\overset{\sim }{A}}_{tr}^{(i)}\right)$$

where ${\hat{Y}}_{tr}^{(i)}\in {\mathbb{R}}^{n_{tr}\times c}$. ${\hat{y}}_j^{(i)}\in {\mathbb{R}}^c$ is denotes the *j*th row in ${\hat{Y}}_{tr}^{(i)}$, which is the predicted label distribution of the *j*th training sample from the *i*th omics data type. Therefore, the loss function for *GCN*_*i*_(·) can be expressed as:

18 $${L}_{GCN}^{(i)}={\sum}_{j=1}^{n_{tr}}{L}_{CE}\left({\hat{y}}_j^{(i)},{y}_j\right)$$

where *L*_*CE*_(·) represents the cross-entropy loss function.

In moGCN, VCDN is also utilized to fuse different types of omics data for classification. For simplicity, this paper first demonstrates how to extend VCDN to accommodate three views. For the predicted label distribution of the *j*th sample from different omics data types ${\hat{y}}_j^{(i)},i=1,2,3$, a cross-omics discovery tensor *C*_*j*_ ∈ *ℝ*^*c* × *c* × *c*^ is constructed, where each entry of *C*_*j*_ can be calculated as:

19 $${C}_{j,{a}_1{a}_2{a}_3}={\hat{y}}_{j,{a}_1}^{(1)}{\hat{y}}_{j,{a}_2}^{(2)}{\hat{y}}_{j,{a}_3}^{(3)}$$

where ${\hat{y}}_{j,a}^{(i)}$ denotes the *a*th entry of ${\hat{y}}_j^{(i)}$.

Then, the obtained tensor *C*_*j*_ is reshaped to a *c*^3^-dimensional vector *c*_*j*_ and is forwarded to *VCDN*(·) for the final prediction. *VCDN*(·) is a fully connected network with the output dimension of *c*. The loss function of *VCDN*(·) can be represented as:

20 $${L}_{VCDN}={\sum}_{j=1}^{n_{tr}}{L}_{CE}\left( VCDN\left({c}_j\right),{y}_j\right)$$

In summary, the total loss function of moGCN can be expressed as:

21 $$L={\sum}_{i=1}^m{L}_{GCN}^{(i)}+\gamma {L}_{VCDN}$$

where *γ* is a trade-off parameter between the omics-specific classification loss and the final classification loss from *VCDN*(·).

### moGAT

This study replaced the GCN model in moGCN with the GAT model to obtain a new model, and except for GCN, other parts of the whole framework remained unchanged. A GAT model also takes two inputs: feature matrix *X* ∈ *R*^*n* × *d*^and adjacency matrix *A* ∈ *R*^*n* × *n*^. Like all attention mechanisms, the GAT calculation also consists of two steps. First, the attention coefficient is calculated. For vertex *i*, the similarity coefficient between its neighbors ($j\in {\mathcal{N}}_i$) and itself is calculated one by one.

22 $${e}_{ij}=a\left(\left[W{h}_i\parallel W{h}_j\right]\right),j\in {\mathcal{N}}_i$$

Linear mapping of a shared parameter *W* adds dimension to the features of the vertex, and this is a common feature augment method. [*Wh*_*i*_ ∥ *Wh*_*j*_] stitches the transformed features of the vertex *i*, *j*; finally, *a*(·) maps the concatenated high-dimensional features to a real number. The attention coefficients are then normalized through *softmax*.

23 $${\alpha}_{ij}=\frac{\exp \left( LeakyReLU\left({e}_{ij}\right)\right)}{\sum_{k\in \mathcal{N}}\mathit{\exp}\left( LeakyReLU\left({e}_{ik}\right)\right)}$$

In the second step, the features are weighted and aggregated according to the calculated attention coefficient.

24 $${h}_i^{\prime }=\sigma \left({\sum}_{j\in \mathcal{N}}{\alpha}_{ij}W{h}_j\right)$$

where ${h}_i^{\prime }$ is the new feature output by GAT for each vertex *i* (fused with neighborhood information).

### Autoencoder

Autoencoder is a deep neural network that copies its input to its output. An autoencoder consists of two parts, i.e., an encoder and a decoder, and both are implemented by neural networks. The encoder and decoder can be expressed in Eq. (25) and Eq. (26), respectively.

25 $$z={f}_{encoder}(x)$$

26 $${x}^{\prime }={f}_{decoder}(z)$$

where *f*_*encoder*_ and *f*_*decoder*_ are multilayer neural networks.

*efAE*: It is similar to *efNN*, and the *p*-omics vectors are concatenated into one feature vector *X*. Therefore, the encoder and the decoder can be represented as *z* = *f*_*encoder*_(*X*) and *X*^′^ = *f*_*decoder*_(*z*), respectively. For the evaluation, *relu* is used for the activation function in all layers of the encoder and the middle layers of the decoder. *tanh* is used in the last layer of the decoder. *tanh* can be expressed in Eq. (27).

27 $$\tanh (z)=\frac{e^z-{e}^{-z}}{e^z+{e}^{-z}}$$

The loss function is the MSE loss *L*_*MSE*_, which can be expressed as:

28 $${L}_{MSE}=\frac{1}{n}{\sum}_{i=1}^n\left({X}^2-{X}^{\prime 2}\right)$$

where *n* is the number of features.

Eventually, the vector *z* is taken as a multi-omics fusion feature.

*lfAE: p* AEs are used to perform feature extraction on the *p*-omics vectors. The encoder and decoder can be expressed in Eq. (29) and Eq. (30), respectively.

29 $${z}_i={f}_{encoder(i)}\left({x}_i\right),i=1,2,\dots, p$$

30 $${x}_i^{\prime }={f}_{decoder(i)}\left({z}_i\right),i=1,2,\dots, p$$

For each AE, this study set the activation and loss functions the same as those in *efAE* in our evaluation. *z*_*i*_ was adopted to represent the latent features of each omics. Finally, the latent features *z*_*i*_ of each omics were concatenated as multi-omics fusion features *z*_*fusion*_.

31 $${z}_{fusion}= concat\left({z}_1,{z}_2,\dots, {z}_p\right)$$

### Denoising autoencoder

Unlike the standard AE, DAE constructs partially damaged data by adding noise to the input data and restores it to the original input data through encoding and decoding. The new generated $\overset{\sim }{x}$ can be expressed in Eq. (32).

32 $$\overset{\sim }{x}={q}_D\left(\overset{\sim }{x}|x\right)$$

where *q*_*D*_ represents the stochastic mapping.

Then, $\overset{\sim }{x}$ is used as the input of the encoder, and *x* is used as the reconstructed target of the decoder. The loss function is consistent with that of the standard AE.

*efDAE:* First, the *p*-omics vectors are concatenated into one feature vector *X*. Then, noise is added to *X* by $\overset{\sim }{X}={q}_D(X)$to obtain $\overset{\sim }{X}$. Next, the encoder and the decoder can be represented as $z={f}_{encoder}\left(\overset{\sim }{X}\right)$ and *X*^′^ = *f*_*decoder*_(*z*), respectively. The following steps are the same as those in *efAE*. Finally, the vector *z* is taken as a multi-omics fusion feature.

*lfDAE*: First, noise is added to *p*-omics vectors *x*_*i*_ by $\tilde{x}_i={q}_D\left({x}_i\right)$ to obtain $\tilde{x}_i$. Then, *p* AEs are used to perform feature extraction on the *p* new vectors with noise. The encoder and decoder can be expressed in Eq. (33) and Eq. (34), respectively.

33 $${z}_i={f}_{encoder(i)}\left(\tilde{x}_i\right),i=1,2,\dots, p$$

34 $${x}_i^{\prime }={f}_{decoder(i)}\left({z}_i\right),i=1,2,\dots, p$$

The following steps are the same as those in *efAE*. Finally, the latent features *z*_*i*_ of each omics are also concatenated as multi-omics fusion features *z*_*fusion*_.

### Variational autoencoder

Compared with AE, VAE has one more constraint. Thus, the latent vectors of VAE follow closely a unit Gaussian distribution. The final hidden layer of the encoder is fully connected to two output layers, which represent the mean *μ* and the standard deviation *σ* in the Gaussian distribution $\mathcal{N}\left(\mu, \sigma \right)$ of the latent variable *z*, given an input sample *x*. To make the sampling step differentiable and suitable for backpropagation, the reparameterization trick in Eq. (35) is applied.

35 $$z=\mu +\sigma \epsilon$$

where *ϵ* is a random variable sampled from the unit normal distribution $\mathcal{N}\left(0,I\right)$.

The loss function in VAE consists of two parts, i.e., the reconstruction loss and the latent loss. The same as AE, the reconstruction loss is the MSE loss. The latent loss measures how well the latent vectors follow the assumed distribution using the Kullback-Liebler divergence (KL).

36 $${D}_{KL}\left(p(x)\parallel q(x)\right)=-{\sum}_xp(x)\log \frac{q(x)}{p(x)}$$

37 $$L_{KL}=D_{KL}\Big(\mathcal N\left(\mu,\sigma\right)\parallel\mathcal N\left(0,I\right)\Big)$$

where *L*_*KL*_ is the KL divergence between the learned distribution and a unit Gaussian distribution.

Therefore, the total loss function can be defined in Eq. (38).

38 $${L}_{total}={L}_{MSE}-{L}_{KL}$$

*efVAE*: It is similar to *efAE*, and the *p*-omics vectors are also concatenated into one feature vector *X*. *X* is used as the input of VAE. The mean vector *μ* and the standard deviation vector *σ* can be obtained from the encoder. Then, the vector *z* can be obtained by sampling using Eq. (37). Eventually, the vector *z* is taken as a multi-omics fusion feature. In our evaluation, the activation and loss functions were set the same as those in *efAE*.

*lfVAE*: Similar to *lfAE*, *p* VAEs are used to perform feature extraction on the *p*-omics vectors. We can obtain the mean vectors *μ*_*i*_(*i* = 1, 2, …, *p*) and the standard deviation vectors *σ*_*i*_(*i* = 1, 2, …, *p*) from the encoders. Then, the vectors *z*_*i*_(*i* = 1, 2, …, *p*) can be obtained by sampling using Eq. (37). Eventually, latent features *z*_*i*_ of each omics are concatenated as multi-omics fusion features *z*_*fusion*_.

*efSVAE*: SVAE is a stacked VAE model. In SVAE, all hidden layers obey a unit Gaussian distribution. Each hidden layer of the encoder fully connects to two output layers, which represent the mean *μ* and the standard deviation *σ* in the Gaussian distribution $\mathcal{N}\left(0,I\right)$. The sampling step is the same as that in VAE. In the evaluation, a multiplier was added to the loss function similar to β-VA E [69]. The total loss can be expressed in Eq. (39):

39 $${L}_{total}={L}_{MSE}+\beta {L}_{KL}$$

where *β* is initially 0 and is gradually increased by *β* = *β* + *k* until its value reaches 1.

The following steps are the same as those in *efVAE*. Eventually, the vector *z* is taken as a multi-omics fusion feature.

*lfSVAE*: Compared with lfVAE, this model just replaces VAE with SVAE. The vectors *z*_*i*_ (*i* = 1, 2, …, *p*) can be obtained by sampling. Finally, the latent features *z*_*i*_ of each omics are concatenated as multi-omics fusion features *z*_*fusion*_.

*efmmdVAE:* Unlike standard VAE, mmdVAE uses Maximum Mean Discrepancy (MMD) in the loss function instead of the Kullback-Liebler divergence (KL). MMD-based regularization term estimates divergence by how “different” the moments of two distributions *p*(*z*) and *q*(*z*) are. This study used the kernel embedding trick to estimate the MMD for two distributions, as shown in Eq. (40):

40 $$MMD\left(p(z)\parallel q(z)\right)={\mathbb{E}}_{p(z),p\left({z}^{\prime}\right)}\left[k\left(z,{z}^{\prime}\right)\right]+{\mathbb{E}}\left[k\left(z,{z}^{\prime}\right)\right]-2{\mathbb{E}}_{p(z),q\left({z}^{\prime}\right)}\left[k\left(z,{z}^{\prime}\right)\right]$$

where *k*(*z*, *z*^′^) can be any universal kernel.

41 $${L}_{MMD}= MMD\left(\mathcal{N}\left(\mu, \sigma \right)\parallel \mathcal{N}\left(0,I\right)\right)$$

The corresponding loss function can be expressed in Eq. (42).

42 $${L}_{total}={L}_{MSE}+{L}_{MMD}$$

In *efmmdVAE*, one VAE is also used to train the omics data. Except for the different loss function from efVAE, other parts are the same. Finally, the vector *z* is taken as a multi-omics fusion feature.

*lfmmdVAE:* It is similar to lfVAE, and p VAEs are used to train the omics data. Finally, the vectors *z*_*i*_ (*i* = 1, 2, …, *p*) can be obtained by sampling and they are concatenated as multi-omics fusion features *z*_*fusion*_.

### Evaluation metrics

First, a few relevant definitions are introduced:

- **TP** (True-Positive) represents the number of samples that are actually positive cases and are determined as positive cases by the classifier.
- **FP** (False-Positive) represents the number of samples that are actually negative cases but are determined as positive cases by the classifier.
- **FN** (False-Negative) represents the number of samples that are actually positive cases but are determined as negative cases by the classifier.
- **TN** (True-Negative) represents the number of samples that are actually negative cases and are determined as negative cases by the classifier.

### Accuracy

Accuracy represents the ratio between the correctly predicted samples and the total samples:

43 $$accuracy=\frac{TP+ TN}{TP+ FP+ TN+ FN}$$

### F1 macro

The macro algorithm calculates Precision and Recall by first calculating Precision and Recall for each category and then taking the average.

44 $${Precision}_i=\frac{T{P}_i}{T{P}_i+F{P}_i}$$

45 $$Precisio{n}_{macro}={\sum}_{i=1}^L\frac{Preciso{n}_i}{\left|L\right|}$$

46 $$Recal{l}_i=\frac{T{P}_i}{T{P}_i+F{N}_i}$$

47 $$Recal{l}_{macro}={\sum}_{i=1}^L\frac{Recal{l}_i}{\left|L\right|}$$

48 $$F1_{macro}=2\cdot\frac{Precision_{macro}\cdot Recall_{macro}}{Precision_{macro}+Recall_{macro}}$$

### F1 weighted

The weighted algorithm is a modified version of the macro algorithm to address the issue that the macro algorithm does not consider the imbalance in the sample. When calculating Precision and Recall, the Precision and Recall of each category are multiplied by the percentage *w*_*i*_ of that category in the total samples.

49 $$Precisio{n}_{weighted}={\sum}_{i=1}^L\frac{Preciso{n}_i\times {w}_i}{\left|L\right|}$$

50 $$Recal{l}_{weighted}={\sum}_{i=1}^L\frac{Recal{l}_i\times {w}_i}{\left|L\right|}$$

51 $$F1_{weighted}=2\cdot\frac{Precision_{weighted}\cdot Recall_{weighted}}{Precision_{weighted}+Recall_{weighted}}$$

### Jaccard index

JI is a statistic used to compare the similarity and diversity between two finite sets *A* and *B*. It is defined by the size of the intersection of the sets and divided by the size of their union. The value of JI is within [0, 1]. The larger value of JI, the higher the similarity.

52 $$JI\left(A,B\right)=\frac{A\cap B}{A\cup B}$$

### C-index

In the cluster *C*_*k*_, there are $\frac{n_k\left({n}_k-1\right)}{2}$ pairs of distinct points. *N*_*W*_ represents the total number of such pairs:

53 $${N}_W=\frac{1}{2}\left({\sum}_{k=1}^K{n}_k^2-N\right)$$

The total number of pairs of distinct points in the dataset is

54 $${N}_T=\frac{N\left(N-1\right)}{2}$$

The C-index is defined as:

55 $$C- index=\frac{S_W-{S}_{min}}{S_{max}-{S}_{min}}$$

where *S*_*W*_ is the sum of the *N*_*W*_ distances between all the pairs of points inside each cluster; *S*_*min*_ is the sum of the *N*_*W*_ smallest distances between all the pairs of points in the whole dataset, and there are *N*_*T*_ such pairs: one takes the sum of the *N*_*W*_ smallest values; *S*_*max*_ is the sum of the *N*_*W*_ largest distances between all the pairs of points in the whole dataset, and there are *N*_*T*_ such pairs: one takes the sum of the *N*_*W*_ largest values.

### Silhouette score

The silhouette score of this sample can be written as:

56 $$Silhouette\ score=\frac{b-a}{\max \left(a,b\right)}$$

For a sample, *a* is the average distance from other samples in the same category, and *b* is the average distance from samples in the nearest different category.

For a sample set, its silhouette score is the average of the silhouette score of all samples. The range of the silhouette score is [−1, 1]. The closer the samples of the same category and the farther the samples of different categories, the higher the value is. A negative value of the silhouette score indicates a poor clustering performance.

### Davies Bouldin score

The Davies Bouldin score calculates the sum of the average distance of any two categories divided by the center distance of two clusters to obtain the maximum value. A lower Davies Bouldin score means a smaller intra-class distance and a larger inter-class distance. The calculation expression is as follows:

57 $${R}_{ij}=\frac{\left({s}_i+{s}_j\right)}{d_{ij}}$$

58 $$Davies\ Bouldin\ score=\frac{1}{k}{\sum}_{i=1}^k\underset{i\ne j}{\max }{R}_{ij}$$

where *s*_*i*_ represents the average distance between each point of a cluster and the centroid of the cluster, and *d*_*ij*_ represents the distance between the centroids of clusters *i* and *j*.

### Selectivity score

The selectivity score can be defined as:

59 $$S=\frac{N_c+{N}_f}{2L}$$

where *N*_*c*_ is the total number of clinical annotations associated with at least one feature, *N*_*f*_ is the total number of features associated with at least one clinical annotation, and *L* is the total number of associations between clinical annotations and features. When each feature is associated with one and only one clinical annotation, the maximum value of S is 1, and vice versa; the minimum value of S is 0.

### Unified score

Based on the benchmarking results, to make our evaluation more objective, we defined “unified score” to unify the results of each indicator as the final comprehensive evaluation indicator. The unified score is referring to the rank aggregation scheme for the Synapse Challenge (10.7303/syn6131484). This score is equal to the sum over all normalized ranking measures, it is defined as

60 $$U=-\sum \ln \left(\frac{r}{N+1}\right)$$

where *r* is the rank of a method for a specific metric (e.g., accuracy, silhouette score, etc.) and *N* is the total number of methods. Thus, higher scores indicate better performance. If one method was evaluated in more than one scenario (e.g., in simulated multi-omics dataset, all methods are evaluated on six scenarios: three cluster number × same size/random size), we used its average unified scores.

## Supplementary Information

Additional file 1: Table S1. Performance of six supervised methods in the condition that the clusters have variable random sizes. Table S2. JI of ten unsupervised methods on simulated datasets. The results are presented as mean value of JIs. Table S3. C-index of ten unsupervised methods on simulated datasets. Table S4. Silhouette score of ten unsupervised methods on simulated datasets. Table S5. Davies Bouldin score of ten unsupervised methods on simulated datasets. Table S6. JI, C-index, silhouette score, and Davies Bouldin score of ten unsupervised methods on single-cell multi-omics datasets. The JI index is presented as mean value of JIs. Table S7. C-index of ten unsupervised methods on cancer benchmark datasets used in clustering task. Table S8. Silhouette scores of ten unsupervised methods on cancer benchmark datasets used in clustering task. Table S9. Davies Bouldin scores of ten unsupervised methods on cancer benchmark datasets used in clustering task. Table S10. Selectivity score of ten unsupervised methods on cancer benchmark datasets used in clustering task (selectivity scores greater than the average are bolded). Figure S1. Data reduction experiment on cancer benchmark datasets used in classification task. Accuracy (a), F1 macro (b), F1 weighted (c) of the six unsupervised methods for classification under 20%, 40%, 60%, 80% of the total samples in the original data, respectively.

Additional file 2. Review History

## Data Availability

All datasets used in this study are publicly available. The simulated multi-omics datasets were generated by the R package *InterSIM*. The single-cell multi-omics datasets are available in our GitHub repository. The benchmark cancer multi-omics datasets were downloaded from http://acgt.cs.tau.ac.il/multi_omic_benchmark/download.html. All code for evaluation in this paper is available. To reproduce the experimental results, the following main libraries need to be installed: Python:3.7.0, R 3.5.1, Tensorflow 1.15.0, Scikit-learn 0.20.0, and Jupyter 1.0.0. All dataset and codes are available at the https://github.com/zhenglinyi/DL-mo [70] (DOI: 10.5281/zenodo.6876344 [71]).
