# Supplementary material for: A benchmark study of deep learning-based multi-omics data fusion methods for cancer
Source: Genome Biol. 2022 Aug 9;23:171. doi: 10.1186/s13059-022-02739-2 (PMC9361561; doi:10.1186/s13059-022-02739-2)
Supplement: Supplementary file 1 — Additional file 1: Table S1. Performance of six supervised methods in the condition that the clusters have variable random sizes. Table S2. JI of ten unsupervised methods on simulated datasets. The results are presented as mean value of JIs. Table S3. C-index of ten unsupervised methods on simulated datasets. Table S4. Silhouette score of ten unsupervised methods on simulated datasets. Table S5. Davies Bouldin score of ten unsupervised methods on simulated datasets. Table S6. JI, C-index, silhouette score, and Davies Bouldin score of ten unsupervised methods on single-cell multi-omics datasets. The JI index is presented as mean value of JIs. Table S7. C-index of ten unsupervised methods on cancer benchmark datasets used in clustering task. Table S8. Silhouette scores of ten unsupervised methods on cancer benchmark datasets used in clustering task. Table S9. Davies Bouldin scores of ten unsupervised methods on cancer benchmark datasets used in clustering task. Table S10. Selectivity score of ten unsupervised methods on cancer benchmark datasets used in clustering task (selectivity scores greater than the average are bolded). Figure S1. Data reduction experiment on cancer benchmark datasets used in classification task. Accuracy (a), F1 macro (b), F1 weighted (c) of the six unsupervised methods for classification under 20%, 40%, 60%, 80% of the total samples in the original data, respectively. [file 13059_2022_2739_MOESM1_ESM.docx]

**Table S1.** Performance of six supervised methods in the condition that the clusters have variable random sizes

| Methods | 5 clusters of random sizes | | | 10 clusters of random sizes | | | 15 clusters of random sizes | | |
| --- | --- | --- | --- | --- | --- | --- | --- | --- | --- |
|  | Accuracy | F1 macro | F1 weighted | Accuracy | F1 macro | F1 weighted | Accuracy | F1 macro | F1 weighted |
| lfNN | **1.0** | **1.0** | **1.0** | 0.900 | 0.825 | 0.871 | 0.860 | 0.716 | 0.817 |
| efNN | **1.0** | **1.0** | **1.0** | **1.0** | **1.0** | **1.0** | **1.0** | **1.0** | **1.0** |
| lfCNN | **1.0** | **1.0** | **1.0** | 0.480 | 0.454 | 0.361 | 0.880 | 0.758 | 0.840 |
| efCNN | **1.0** | **1.0** | **1.0** | **1.0** | **1.0** | **1.0** | 0.760 | 0.572 | 0.670 |
| moGCN | **1.0** | **1.0** | **1.0** | **1.0** | **1.0** | **1.0** | **1.0** | **1.0** | **1.0** |
| moGAT | **1.0** | **1.0** | **1.0** | **1.0** | **1.0** | **1.0** | **1.0** | **1.0** | **1.0** |

**Table S2.** JI of ten unsupervised methods on simulated datasets. The results are presented as mean value of JIs.

| Methods | Same size | | | Random sizes | | | |
| --- | --- | --- | --- | --- | --- | --- | --- |
|  | 5 clusters | 10 clusters | 15 clusters | 5 clusters | 10 clusters | 15 clusters |  |
| lfAE | 0.764 | 0.744 | 0.759 | 0.708 | 0.729 | 0.712 |  |
| efAE | **0.895** | **0.868** | **0.863** | **0.886** | **0.834** | **0.788** |  |
| lfDAE | 0.784 | 0.737 | 0.746 | 0.755 | 0.716 | 0.649 |  |
| efDAE | 0.871 | 0.850 | 0.822 | 0.765 | 0.793 | 0.762 |  |
| lfVAE | 0.805 | 0.715 | 0.756 | 0.598 | 0.672 | 0.571 |  |
| efVAE | 0.815 | 0.780 | 0.766 | 0.824 | 0.684 | 0.602 |  |
| lfSVAE | 0.186 | 0.170 | 0.182 | 0.195 | 0.155 | 0.168 |  |
| efSVAE | 0.201 | 0.160 | 0.176 | 0.203 | 0.150 | 0.156 |  |
| lfmmdVAE | 0.839 | 0.734 | 0.757 | 0.843 | 0.694 | 0.617 |  |
| efmmdVAE | 0.691 | 0.745 | 0.724 | 0.691 | 0.711 | 0.651 |  |

**Table S3.** C-index of ten unsupervised methods on simulated datasets.

| Methods | Same size | | | Random sizes | | |
| --- | --- | --- | --- | --- | --- | --- |
|  | 5 clusters | 10 clusters | 15 clusters | 5 clusters | 10 clusters | 15 clusters |
| lfAE | **0.032** | 0.068 | 0.055 | 0.041 | 0.062 | 0.074 |
| efAE | 0.057 | 0.058 | 0.055 | 0.047 | 0.065 | 0.057 |
| lfDAE | 0.060 | 0.057 | 0.063 | 0.045 | 0.068 | 0.095 |
| efDAE | 0.051 | 0.059 | 0.056 | 0.083 | 0.073 | 0.080 |
| lfVAE | 0.052 | 0.046 | 0.052 | 0.046 | 0.059 | 0.048 |
| efVAE | 0.071 | 0.037 | **0.041** | 0.068 | 0.087 | 0.066 |
| lfSVAE | 0.202 | 0.178 | 0.144 | 0.173 | 0.165 | 0.160 |
| efSVAE | 0.216 | 0.143 | 0.167 | 0.195 | 0.161 | 0.151 |
| lfmmdVAE | 0.038 | **0.034** | 0.025 | **0.023** | **0.051** | **0.047** |
| efmmdVAE | 0.127 | 0.054 | 0.064 | 0.092 | 0.053 | 0.052 |

**Table S4.** Silhouette score of ten unsupervised methods on simulated datasets.

| Methods | Same size | | | Random sizes | | |
| --- | --- | --- | --- | --- | --- | --- |
|  | 5 clusters | 10 clusters | 15 clusters | 5 clusters | 10 clusters | 15 clusters |
| lfAE | 0.728 | 0.691 | **0.707** | 0.739 | 0.715 | 0.715 |
| efAE | 0.468 | 0.427 | 0.428 | 0.507 | 0.462 | 0.384 |
| lfDAE | 0.716 | **0.719** | 0.704 | 0.671 | 0.753 | 0.715 |
| efDAE | 0.612 | 0.572 | 0.610 | 0.606 | 0.616 | 0.591 |
| lfVAE | 0.796 | 0.600 | 0.585 | 0.452 | 0.571 | 0.541 |
| efVAE | **0.825** | 0.686 | 0.632 | **0.831** | **0.826** | **0.751** |
| lfSVAE | 0.165 | 0.098 | 0.074 | 0.174 | 0.095 | 0.069 |
| efSVAE | 0.166 | 0.117 | 0.068 | 0.180 | 0.103 | 0.072 |
| lfmmdVAE | 0.785 | 0.699 | 0.631 | 0.655 | 0.707 | 0.651 |
| efmmdVAE | 0.285 | 0.315 | 0.243 | 0.303 | 0.303 | 0.319 |

**Table S5.** Davies Bouldin score of ten unsupervised methods on simulated datasets.

| Methods | Same size | | | Random sizes | | |
| --- | --- | --- | --- | --- | --- | --- |
|  | 5 clusters | 10 clusters | 15 clusters | 5 clusters | 10 clusters | 15 clusters |
| lfAE | 0.389 | 0.475 | **0.408** | 0.414 | 0.366 | 0.391 |
| efAE | 0.828 | 0.869 | 0.856 | 0.817 | 0.798 | 0.977 |
| lfDAE | 0.430 | **0.423** | 0.424 | 0.480 | 0.329 | 0.406 |
| efDAE | 0.564 | 0.664 | 0.560 | 0.546 | 0.524 | 0.595 |
| lfVAE | 0.290 | 0.614 | 0.587 | 0.774 | 0.712 | 0.760 |
| efVAE | **0.261** | 0.423 | 0.517 | **0.236** | **0.240** | **0.371** |
| lfSVAE | 1.511 | 1.737 | 1.717 | 1.506 | 1.675 | 1.722 |
| efSVAE | 1.523 | 1.628 | 1.726 | 1.432 | 1.671 | 1.700 |
| lfmmdVAE | 0.288 | 0.424 | 0.518 | 0.492 | 0.431 | 0.544 |
| efmmdVAE | 1.199 | 1.136 | 1.238 | 1.149 | 1.182 | 1.142 |

**Table S6.** JI, C-index, silhouette score, and Davies Bouldin score of ten unsupervised methods on single-cell multi-omics datasets. The JI index is presented as mean value of JIs.

| Methods | Mean value of JIs ↑ | C-index ↓ | Silhouette score ↑ | Davies Bouldin score ↓ |
| --- | --- | --- | --- | --- |
| lfAE | 0.291 | **0.048** | **0.684** | 0.483 |
| efAE | 0.331 | 0.049 | 0.405 | 0.810 |
| lfDAE | 0.366 | 0.054 | 0.600 | **0.471** |
| efDAE | 0.441 | 0.068 | 0.476 | 0.706 |
| lfVAE | 0.511 | 0.050 | 0.534 | 0.598 |
| efVAE | 0.548 | 0.078 | 0.486 | 0.800 |
| lfSVAE | 0.217 | 0.165 | 0.332 | 1.013 |
| efSVAE | 0.382 | 0.084 | 0.425 | 0.805 |
| lfmmdVAE | 0.202 | 0.084 | 0.435 | 0.760 |
| efmmdVAE | **0.564** | 0.135 | 0.587 | 0.512 |

**Table S7.** C-index of ten unsupervised methods on cancer benchmark datasets used in clustering task

| Methods | Number of clusters | AML | BRCA | COAD | GBM | LIHC | LUSC | SKCM | OV | SARC | KIRC |  |
| --- | --- | --- | --- | --- | --- | --- | --- | --- | --- | --- | --- | --- |
|  |  |  |  |  |  |  |  |  |  |  |  |  |
| **lfAE** | 2 | 0.221 | 0.219 | 0.180 | 0.244 | 0.149 | 0.262 | 0.263 | 0.275 | 0.216 | 0.251 |  |
|  | 3 | 0.177 | 0.202 | 0.163 | 0.211 | 0.079 | 0.223 | 0.198 | 0.225 | 0.174 | 0.178 |  |
|  | 4 | 0.145 | 0.180 | 0.131 | 0.180 | 0.106 | 0.198 | 0.174 | 0.192 | 0.155 | 0.140 |  |
|  | 5 | 0.140 | 0.157 | 0.134 | 0.166 | 0.107 | 0.181 | 0.160 | 0.179 | 0.137 | 0.134 |  |
|  | 6 | 0.133 | 0.149 | 0.133 | 0.154 | 0.104 | 0.168 | 0.140 | 0.170 | 0.124 | 0.127 |  |
| **efAE** | 2 | 0.312 | 0.307 | 0.244 | 0.305 | 0.194 | 0.277 | 0.258 | 0.299 | 0.308 | 0.283 |  |
|  | 3 | 0.259 | 0.264 | 0.239 | 0.252 | 0.167 | 0.271 | 0.255 | 0.266 | 0.251 | 0.227 |  |
|  | 4 | 0.228 | 0.242 | 0.222 | 0.221 | 0.154 | 0.257 | 0.246 | 0.248 | 0.213 | 0.212 |  |
|  | 5 | 0.209 | 0.228 | 0.208 | 0.197 | 0.160 | 0.244 | 0.231 | 0.234 | 0.202 | 0.202 |  |
|  | 6 | 0.195 | 0.218 | 0.198 | 0.185 | 0.158 | 0.231 | 0.223 | 0.221 | 0.187 | 0.192 |  |
| **lfDAE** | 2 | 0.238 | 0.170 | 0.112 | 0.262 | 0.191 | 0.213 | 0.183 | 0.254 | 0.208 | 0.191 |  |
|  | 3 | 0.154 | 0.166 | 0.150 | 0.173 | 0.143 | 0.188 | 0.138 | 0.219 | 0.177 | 0.158 |  |
|  | 4 | 0.144 | 0.128 | 0.135 | 0.149 | 0.116 | 0.177 | 0.130 | 0.199 | 0.156 | 0.119 |  |
|  | 5 | 0.128 | 0.139 | 0.114 | 0.138 | 0.109 | 0.169 | 0.123 | 0.181 | 0.134 | 0.110 |  |
|  | 6 | 0.117 | 0.131 | 0.101 | 0.126 | 0.111 | 0.160 | 0.118 | 0.165 | 0.122 | 0.104 |  |
| **efDAE** | 2 | 0.276 | 0.294 | 0.231 | 0.286 | 0.218 | 0.290 | 0.303 | 0.268 | 0.281 | 0.232 |  |
|  | 3 | 0.235 | 0.262 | 0.222 | 0.242 | 0.201 | 0.260 | 0.242 | 0.253 | 0.237 | 0.199 |  |
|  | 4 | 0.210 | 0.246 | 0.204 | 0.213 | 0.183 | 0.234 | 0.221 | 0.232 | 0.208 | 0.156 |  |
|  | 5 | 0.194 | 0.235 | 0.186 | 0.189 | 0.174 | 0.221 | 0.210 | 0.216 | 0.188 | 0.152 |  |
|  | 6 | 0.182 | 0.224 | 0.180 | 0.173 | 0.166 | 0.212 | 0.202 | 0.206 | 0.173 | 0.143 |  |
| **lfVAE** | 2 | 0.254 | 0.216 | 0.182 | 0.123 | 0.080 | 0.204 | 0.221 | 0.300 | 0.160 | 0.175 |  |
|  | 3 | 0.184 | 0.161 | 0.155 | 0.050 | 0.095 | 0.177 | 0.191 | 0.214 | 0.116 | 0.079 |  |
|  | 4 | 0.140 | 0.098 | 0.131 | 0.089 | 0.081 | 0.160 | 0.175 | 0.192 | 0.103 | 0.089 |  |
|  | 5 | 0.111 | 0.105 | 0.118 | 0.097 | 0.073 | 0.138 | 0.156 | 0.172 | 0.093 | 0.093 |  |
|  | 6 | 0.093 | 0.104 | 0.108 | 0.091 | 0.067 | 0.121 | 0.147 | 0.155 | 0.094 | 0.091 |  |
| **efVAE** | 2 | 0.136 | 0.176 | 0.155 | 0.086 | 0.077 | 0.193 | 0.253 | 0.281 | 0.042 | 0.143 |  |
|  | 3 | 0.086 | 0.135 | 0.136 | 0.109 | 0.077 | 0.154 | 0.215 | 0.223 | 0.067 | 0.044 |  |
|  | 4 | 0.041 | 0.088 | 0.112 | 0.095 | 0.076 | 0.119 | 0.181 | 0.186 | 0.060 | 0.075 |  |
|  | 5 | 0.042 | 0.086 | 0.094 | 0.078 | 0.064 | 0.107 | 0.160 | 0.167 | 0.051 | 0.085 |  |
|  | 6 | 0.039 | 0.085 | 0.079 | 0.068 | 0.059 | 0.098 | 0.147 | 0.153 | 0.045 | 0.092 |  |
| **lfSVAE** | 2 | 0.370 | 0.369 | 0.366 | 0.359 | 0.369 | 0.393 | 0.333 | 0.384 | 0.372 | 0.369 |  |
|  | 3 | 0.328 | 0.313 | 0.317 | 0.269 | 0.253 | 0.338 | 0.299 | 0.326 | 0.309 | 0.316 |  |
|  | 4 | 0.296 | 0.289 | 0.290 | 0.231 | 0.209 | 0.305 | 0.273 | 0.293 | 0.276 | 0.276 |  |
|  | 5 | 0.269 | 0.267 | 0.269 | 0.212 | 0.200 | 0.280 | 0.258 | 0.269 | 0.248 | 0.247 |  |
|  | 6 | 0.251 | 0.249 | 0.251 | 0.197 | 0.188 | 0.260 | 0.245 | 0.252 | 0.227 | 0.228 |  |
| **efSVAE** | 2 | 0.356 | 0.211 | 0.097 | 0.371 | 0.047 | 0.226 | 0.224 | 0.203 | 0.114 | 0.350 |  |
|  | 3 | 0.306 | 0.161 | 0.076 | 0.326 | 0.039 | 0.157 | 0.210 | 0.151 | 0.124 | 0.310 |  |
|  | 4 | 0.277 | 0.134 | 0.076 | 0.295 | 0.042 | 0.133 | 0.179 | 0.140 | 0.104 | 0.281 |  |
|  | 5 | 0.256 | 0.115 | 0.076 | 0.274 | 0.041 | 0.112 | 0.163 | 0.124 | 0.086 | 0.256 |  |
|  | 6 | 0.238 | 0.102 | 0.075 | 0.259 | 0.038 | 0.095 | 0.152 | 0.118 | 0.079 | 0.240 |  |
| **lfmmdVAE** | 2 | 0.125 | 0.133 | 0.136 | 0.218 | 0.048 | 0.227 | 0.192 | 0.327 | 0.275 | 0.174 |  |
|  | 3 | 0.092 | 0.127 | 0.153 | 0.126 | 0.048 | 0.206 | 0.192 | 0.271 | 0.163 | 0.116 |  |
|  | 4 | 0.090 | 0.107 | 0.134 | 0.106 | 0.043 | 0.184 | 0.166 | 0.238 | 0.149 | 0.111 |  |
|  | 5 | 0.086 | 0.103 | 0.123 | 0.100 | 0.050 | 0.162 | 0.151 | 0.213 | 0.137 | 0.105 |  |
|  | 6 | 0.082 | 0.096 | 0.113 | 0.086 | 0.046 | 0.148 | 0.134 | 0.194 | 0.127 | 0.103 |  |
| **efmmdVAE** | 2 | 0.140 | 0.132 | 0.072 | 0.186 | 0.108 | 0.113 | 0.187 | 0.133 | 0.063 | 0.032 |  |
|  | 3 | 0.098 | 0.139 | 0.089 | 0.093 | 0.118 | 0.100 | 0.163 | 0.074 | 0.064 | 0.011 |  |
|  | 4 | 0.076 | 0.110 | 0.068 | 0.043 | 0.106 | 0.083 | 0.130 | 0.055 | 0.081 | 0.033 |  |
|  | 5 | 0.069 | 0.116 | 0.060 | 0.040 | 0.083 | 0.080 | 0.112 | 0.052 | 0.083 | 0.045 |  |
|  | 6 | 0.063 | 0.105 | 0.050 | 0.035 | 0.080 | 0.073 | 0.104 | 0.052 | 0.081 | 0.044 |  |

**Table S8.** Silhouette scores of ten unsupervised methods on cancer benchmark datasets used in clustering task

| Methods | Number of clusters | AML | KIRC | LIHC | SARC | GBM | BRCA | COAD | LUSC | SKCM | OV |
| --- | --- | --- | --- | --- | --- | --- | --- | --- | --- | --- | --- |
| **lfAE** | 2 | 0.21 | 0.19 | 0.32 | 0.21 | 0.23 | 0.22 | 0.24 | 0.18 | 0.19 | 0.17 |
|  | 3 | 0.21 | 0.21 | 0.34 | 0.21 | 0.20 | 0.19 | 0.21 | 0.17 | 0.19 | 0.17 |
|  | 4 | 0.21 | 0.22 | 0.27 | 0.20 | 0.19 | 0.19 | 0.19 | 0.16 | 0.19 | 0.17 |
|  | 5 | 0.21 | 0.21 | 0.24 | 0.21 | 0.18 | 0.19 | 0.18 | 0.16 | 0.17 | 0.16 |
|  | 6 | 0.21 | 0.20 | 0.23 | 0.21 | 0.19 | 0.17 | 0.17 | 0.15 | 0.18 | 0.16 |
| **efAE** | 2 | 0.13 | 0.15 | 0.20 | 0.13 | 0.15 | 0.12 | 0.15 | 0.14 | 0.14 | 0.13 |
|  | 3 | 0.13 | 0.15 | 0.19 | 0.13 | 0.14 | 0.12 | 0.12 | 0.10 | 0.10 | 0.12 |
|  | 4 | 0.13 | 0.14 | 0.18 | 0.15 | 0.15 | 0.12 | 0.12 | 0.10 | 0.10 | 0.11 |
|  | 5 | 0.13 | 0.14 | 0.15 | 0.14 | 0.15 | 0.11 | 0.12 | 0.10 | 0.11 | 0.11 |
|  | 6 | 0.13 | 0.14 | 0.16 | 0.14 | 0.15 | 0.11 | 0.12 | 0.10 | 0.10 | 0.11 |
| **lfDAE** | 2 | 0.20 | 0.24 | 0.27 | 0.23 | 0.23 | 0.28 | 0.33 | 0.23 | 0.28 | 0.19 |
|  | 3 | 0.22 | 0.27 | 0.28 | 0.22 | 0.24 | 0.22 | 0.20 | 0.19 | 0.25 | 0.17 |
|  | 4 | 0.22 | 0.26 | 0.27 | 0.22 | 0.24 | 0.23 | 0.20 | 0.16 | 0.20 | 0.17 |
|  | 5 | 0.21 | 0.24 | 0.26 | 0.23 | 0.23 | 0.21 | 0.21 | 0.15 | 0.20 | 0.16 |
|  | 6 | 0.21 | 0.23 | 0.24 | 0.24 | 0.23 | 0.20 | 0.20 | 0.15 | 0.18 | 0.17 |
| **efDAE** | 2 | 0.15 | 0.18 | 0.18 | 0.15 | 0.16 | 0.13 | 0.18 | 0.13 | 0.13 | 0.15 |
|  | 3 | 0.14 | 0.19 | 0.16 | 0.15 | 0.15 | 0.11 | 0.15 | 0.11 | 0.13 | 0.13 |
|  | 4 | 0.15 | 0.20 | 0.16 | 0.15 | 0.15 | 0.11 | 0.15 | 0.11 | 0.12 | 0.12 |
|  | 5 | 0.15 | 0.19 | 0.16 | 0.15 | 0.17 | 0.11 | 0.14 | 0.11 | 0.12 | 0.12 |
|  | 6 | 0.14 | 0.18 | 0.16 | 0.15 | 0.16 | 0.10 | 0.13 | 0.11 | 0.12 | 0.12 |
| **lfVAE** | 2 | 0.22 | 0.37 | 0.42 | 0.34 | 0.41 | 0.28 | 0.29 | 0.28 | 0.24 | 0.19 |
|  | 3 | 0.24 | 0.34 | 0.38 | 0.32 | 0.46 | 0.30 | 0.26 | 0.22 | 0.21 | 0.20 |
|  | 4 | 0.27 | 0.30 | 0.29 | 0.29 | 0.34 | 0.28 | 0.25 | 0.20 | 0.20 | 0.19 |
|  | 5 | 0.28 | 0.29 | 0.30 | 0.29 | 0.30 | 0.24 | 0.23 | 0.20 | 0.19 | 0.18 |
|  | 6 | 0.31 | 0.28 | 0.28 | 0.27 | 0.29 | 0.23 | 0.23 | 0.19 | 0.17 | 0.19 |
| **efVAE** | 2 | 0.50 | 0.44 | 0.48 | 0.58 | 0.52 | 0.32 | 0.39 | 0.36 | 0.22 | 0.22 |
|  | 3 | 0.45 | 0.45 | 0.35 | 0.38 | 0.33 | 0.25 | 0.31 | 0.29 | 0.19 | 0.20 |
|  | 4 | 0.42 | 0.36 | 0.36 | 0.33 | 0.32 | 0.30 | 0.30 | 0.27 | 0.20 | 0.21 |
|  | 5 | 0.36 | 0.36 | 0.31 | 0.32 | 0.29 | 0.25 | 0.31 | 0.26 | 0.21 | 0.21 |
|  | 6 | 0.32 | 0.33 | 0.29 | 0.33 | 0.29 | 0.24 | 0.31 | 0.23 | 0.20 | 0.19 |
| **lfSVAE** | 2 | 0.10 | 0.11 | 0.12 | 0.49 | 0.15 | 0.11 | 0.10 | 0.09 | 0.13 | 0.09 |
|  | 3 | 0.09 | 0.09 | 0.15 | 0.10 | 0.13 | 0.10 | 0.09 | 0.08 | 0.10 | 0.09 |
|  | 4 | 0.09 | 0.09 | 0.11 | 0.09 | 0.11 | 0.08 | 0.09 | 0.08 | 0.09 | 0.09 |
|  | 5 | 0.09 | 0.09 | 0.09 | 0.09 | 0.10 | 0.08 | 0.09 | 0.07 | 0.09 | 0.09 |
|  | 6 | 0.09 | 0.09 | 0.09 | 0.09 | 0.09 | 0.08 | 0.09 | 0.07 | 0.08 | 0.08 |
| **efSVAE** | 2 | 0.11 | 0.11 | 0.55 | 0.37 | 0.09 | 0.27 | 0.39 | 0.27 | 0.22 | 0.27 |
|  | 3 | 0.10 | 0.09 | 0.42 | 0.28 | 0.09 | 0.26 | 0.26 | 0.26 | 0.17 | 0.18 |
|  | 4 | 0.09 | 0.09 | 0.33 | 0.25 | 0.09 | 0.27 | 0.19 | 0.21 | 0.17 | 0.16 |
|  | 5 | 0.09 | 0.09 | 0.29 | 0.26 | 0.09 | 0.27 | 0.18 | 0.21 | 0.18 | 0.14 |
|  | 6 | 0.09 | 0.09 | 0.28 | 0.24 | 0.09 | 0.26 | 0.15 | 0.21 | 0.18 | 0.13 |
| **lfmmdVAE** | 2 | 0.39 | 0.47 | 0.53 | 0.39 | 0.34 | 0.50 | 0.41 | 0.48 | 0.39 | 0.35 |
|  | 3 | 0.29 | 0.48 | 0.45 | 0.31 | 0.37 | 0.43 | 0.39 | 0.39 | 0.29 | 0.28 |
|  | 4 | 0.30 | 0.40 | 0.44 | 0.29 | 0.30 | 0.38 | 0.34 | 0.32 | 0.27 | 0.24 |
|  | 5 | 0.29 | 0.38 | 0.41 | 0.28 | 0.27 | 0.38 | 0.36 | 0.31 | 0.25 | 0.23 |
|  | 6 | 0.29 | 0.36 | 0.38 | 0.27 | 0.29 | 0.37 | 0.35 | 0.28 | 0.26 | 0.22 |
| **efmmdVAE** | 2 | 0.37 | 0.59 | 0.35 | 0.42 | 0.47 | 0.34 | 0.45 | 0.38 | 0.25 | 0.42 |
|  | 3 | 0.38 | 0.64 | 0.33 | 0.40 | 0.45 | 0.33 | 0.40 | 0.35 | 0.24 | 0.33 |
|  | 4 | 0.36 | 0.51 | 0.32 | 0.39 | 0.45 | 0.32 | 0.40 | 0.32 | 0.24 | 0.27 |
|  | 5 | 0.33 | 0.47 | 0.34 | 0.33 | 0.40 | 0.30 | 0.37 | 0.29 | 0.25 | 0.22 |
|  | 6 | 0.31 | 0.41 | 0.33 | 0.30 | 0.39 | 0.30 | 0.37 | 0.28 | 0.25 | 0.21 |

**Table S9.** Davies Bouldin scores of ten unsupervised methods on cancer benchmark datasets used in clustering task

| Methods | Number of clusters | AML | KIRC | LIHC | SARC | GBM | BRCA | COAD | LUSC | SKCM | | OV | |
| --- | --- | --- | --- | --- | --- | --- | --- | --- | --- | --- | --- | --- | --- |
| **lfAE** | 2 | 1.74 | 1.92 | 1.39 | 1.80 | 1.75 | 1.87 | 1.62 | 1.95 | 1.96 | 2.03 | |  |
|  | 3 | 1.66 | 1.59 | 1.04 | 1.59 | 1.59 | 1.69 | 1.54 | 1.78 | 1.76 | 1.80 | |  |
|  | 4 | 1.56 | 1.45 | 1.50 | 1.49 | 1.49 | 1.65 | 1.56 | 1.70 | 1.62 | 1.64 | |  |
|  | 5 | 1.48 | 1.56 | 1.49 | 1.36 | 1.43 | 1.53 | 1.68 | 1.66 | 1.64 | 1.68 | |  |
|  | 6 | 1.43 | 1.54 | 1.42 | 1.39 | 1.45 | 1.49 | 1.68 | 1.66 | 1.59 | 1.68 | |  |
| **efAE** | 2 | 2.47 | 2.31 | 2.09 | 2.55 | 2.32 | 2.58 | 2.31 | 2.45 | 2.55 | 2.55 | |  |
|  | 3 | 2.09 | 2.07 | 2.00 | 2.11 | 2.04 | 2.38 | 2.18 | 2.47 | 2.40 | 2.27 | |  |
|  | 4 | 1.89 | 1.87 | 1.98 | 1.85 | 1.90 | 2.20 | 2.05 | 2.25 | 2.24 | 2.15 | |  |
|  | 5 | 1.82 | 1.88 | 1.99 | 1.80 | 1.77 | 2.12 | 1.96 | 2.18 | 2.06 | 2.05 | |  |
|  | 6 | 1.79 | 1.92 | 1.97 | 1.72 | 1.72 | 2.04 | 1.89 | 2.10 | 1.99 | 1.97 | |  |
| **lfDAE** | 2 | 1.88 | 1.66 | 1.56 | 1.74 | 1.64 | 1.58 | 1.26 | 1.73 | 1.60 | 1.90 | |  |
|  | 3 | 1.53 | 1.38 | 1.24 | 1.58 | 1.34 | 1.52 | 1.69 | 1.72 | 1.52 | 1.76 | |  |
|  | 4 | 1.55 | 1.37 | 1.32 | 1.51 | 1.29 | 1.49 | 1.58 | 1.71 | 1.54 | 1.70 | |  |
|  | 5 | 1.46 | 1.46 | 1.38 | 1.31 | 1.29 | 1.43 | 1.40 | 1.69 | 1.52 | 1.65 | |  |
|  | 6 | 1.45 | 1.42 | 1.42 | 1.29 | 1.29 | 1.42 | 1.48 | 1.65 | 1.54 | 1.53 | |  |
| **efDAE** | 2 | 2.29 | 2.02 | 2.07 | 2.24 | 2.22 | 2.60 | 2.09 | 2.55 | 2.43 | 2.19 | |  |
|  | 3 | 1.96 | 1.68 | 2.04 | 1.96 | 1.94 | 2.46 | 1.99 | 2.37 | 2.15 | 2.10 | |  |
|  | 4 | 1.72 | 1.66 | 1.96 | 1.76 | 1.76 | 2.25 | 1.83 | 2.13 | 2.07 | 2.03 | |  |
|  | 5 | 1.80 | 1.65 | 1.90 | 1.66 | 1.66 | 2.13 | 1.80 | 2.12 | 1.97 | 1.94 | |  |
|  | 6 | 1.75 | 1.70 | 1.92 | 1.66 | 1.61 | 2.04 | 1.77 | 2.09 | 1.97 | 1.88 | |  |
| **lfVAE** | 2 | 1.79 | 1.16 | 0.95 | 1.25 | 1.13 | 1.38 | 1.38 | 1.37 | 1.68 | 1.88 | |  |
|  | 3 | 1.46 | 1.15 | 0.97 | 1.26 | 0.93 | 1.16 | 1.39 | 1.59 | 1.56 | 1.50 | |  |
|  | 4 | 1.37 | 1.18 | 1.25 | 1.23 | 1.05 | 1.19 | 1.35 | 1.61 | 1.52 | 1.51 | |  |
|  | 5 | 1.20 | 1.31 | 1.25 | 1.27 | 1.14 | 1.40 | 1.27 | 1.49 | 1.56 | 1.49 | |  |
|  | 6 | 1.02 | 1.32 | 1.23 | 1.28 | 1.16 | 1.38 | 1.30 | 1.44 | 1.54 | 1.44 | |  |
| **efVAE** | 2 | 0.69 | 0.88 | 0.78 | 0.60 | 0.82 | 1.16 | 1.00 | 1.03 | 1.74 | 1.62 | |  |
|  | 3 | 0.69 | 0.88 | 1.17 | 1.01 | 1.03 | 1.35 | 1.14 | 1.09 | 1.67 | 1.57 | |  |
|  | 4 | 0.75 | 0.96 | 1.01 | 1.09 | 1.05 | 1.13 | 1.04 | 1.15 | 1.48 | 1.38 | |  |
|  | 5 | 0.86 | 1.03 | 1.06 | 1.06 | 1.05 | 1.32 | 1.04 | 1.14 | 1.38 | 1.33 | |  |
|  | 6 | 0.93 | 1.15 | 1.10 | 1.06 | 1.05 | 1.36 | 1.04 | 1.25 | 1.42 | 1.38 | |  |
| **lfSVAE** | 2 | 2.77 | 2.64 | 2.27 | 1.21 | 2.11 | 2.69 | 2.70 | 2.92 | 2.46 | 2.84 | |  |
|  | 3 | 2.53 | 2.42 | 2.12 | 2.04 | 2.00 | 2.39 | 2.46 | 2.56 | 2.39 | 2.47 | |  |
|  | 4 | 2.34 | 2.29 | 2.01 | 1.88 | 2.09 | 2.41 | 2.33 | 2.44 | 2.27 | 2.35 | |  |
|  | 5 | 2.21 | 2.12 | 2.08 | 1.88 | 2.01 | 2.33 | 2.23 | 2.39 | 2.30 | 2.26 | |  |
|  | 6 | 2.11 | 2.09 | 1.99 | 1.87 | 2.00 | 2.26 | 2.13 | 2.27 | 2.30 | 2.18 | |  |
| **efSVAE** | 2 | 2.60 | 2.63 | 0.65 | 1.08 | 2.85 | 1.38 | 0.96 | 1.36 | 1.77 | 1.33 | |  |
|  | 3 | 2.37 | 2.39 | 0.90 | 1.30 | 2.53 | 1.31 | 1.26 | 1.21 | 1.78 | 1.59 | |  |
|  | 4 | 2.21 | 2.32 | 1.08 | 1.30 | 2.36 | 1.27 | 1.57 | 1.32 | 1.64 | 1.73 | |  |
|  | 5 | 2.20 | 2.24 | 1.20 | 1.22 | 2.20 | 1.22 | 1.67 | 1.30 | 1.51 | 1.77 | |  |
|  | 6 | 2.11 | 2.15 | 1.18 | 1.25 | 2.15 | 1.26 | 1.75 | 1.33 | 1.50 | 1.81 | |  |
| **lfmmdVAE** | 2 | 0.96 | 0.88 | 0.64 | 0.99 | 1.13 | 0.71 | 0.90 | 0.74 | 0.97 | 1.04 | |  |
|  | 3 | 1.14 | 0.74 | 0.70 | 1.15 | 0.96 | 0.77 | 0.91 | 0.85 | 1.18 | 1.14 | |  |
|  | 4 | 1.11 | 0.89 | 0.69 | 1.09 | 1.04 | 0.87 | 0.90 | 0.95 | 1.19 | 1.24 | |  |
|  | 5 | 1.09 | 0.94 | 0.72 | 1.12 | 1.12 | 0.87 | 0.84 | 0.97 | 1.27 | 1.27 | |  |
|  | 6 | 1.06 | 0.93 | 0.79 | 1.13 | 1.04 | 0.89 | 0.87 | 0.28 | 1.24 | 1.24 | |  |
| **efmmdVAE** | 2 | 1.12 | 0.63 | 1.24 | 1.00 | 0.77 | 1.38 | 0.91 | 1.11 | 1.59 | 0.85 | |  |
|  | 3 | 0.93 | 0.54 | 1.27 | 1.17 | 0.72 | 1.15 | 0.95 | 1.08 | 1.42 | 0.99 | |  |
|  | 4 | 0.92 | 0.73 | 1.15 | 0.99 | 0.69 | 1.09 | 0.87 | 1.10 | 1.39 | 1.12 | |  |
|  | 5 | 0.96 | 0.78 | 1.02 | 1.12 | 0.78 | 1.19 | 0.90 | 1.16 | 1.29 | 1.29 | |  |
|  | 6 | 1.00 | 0.92 | 1.05 | 1.19 | 0.79 | 1.13 | 0.89 | 1.15 | 1.25 | 1.40 | |  |

**Table S10.** Selectivity score of ten unsupervised methods on cancer benchmark datasets used in clustering task (selectivity scores greater than the average are bolded).

|  | lfAE | efAE | lfDAE | efDAE | lfVAE | efVAE | lfSVAE | efSVAE | lfmmdVAE | efmmdVAE |
| --- | --- | --- | --- | --- | --- | --- | --- | --- | --- | --- |
| AML | 0.45 | **0.5** | **0.5** | 0.353 | 0.417 | **0.5** | 1 | **1** | **0.5** | 0.382 |
| BRCA | **0.5** | **0.6** | **0.583** | **0.5** | 0.438 | 0.385 | 0.444 | 0.444 | 0.324 | **0.5** |
| COAD | **0.625** | **0.571** | **0.75** | **0.563** | 0.6 | **0.625** | **0.75** | **0.75** | **0.625** | **0.75** |
| GBM | **0.5** | 0.367 | 0.455 | 0.389 | 0.433 | 0.406 | **0.833** | **0.833** | 0.458 | 0.342 |
| LIHC | 0.462 | 0.458 | 0.393 | 0.462 | 0.316 | 0.429 | 0.353 | 0.353 | 0.455 | 0.45 |
| LUSC | **0.5** | **0.5** | **0.556** | 0.455 | 0.45 | 0.409 | **0.5** | **0.5** | **0.583** | **0.545** |
| SKCM | **0.563** | **0.5** | **0.8** | 0.393 | 0.7 | 0.455 | **0.6** | **0.6** | **0.833** | **0.625** |
| OV | **0.583** | **0.6** | **0.571** | **0.563** | **0.583** | **0.583** | **1** | **1** | **0.625** | **0.75** |
| SARC | 0.429 | 0.464 | 0.367 | 0.4 | 0.406 | 0.31 | 0.375 | 0.375 | **0.8** | 0.429 |
| KIRC | 0 | 0 | 0 | 0 | 0 | 0 | 0 | 0 | 0 | 0 |


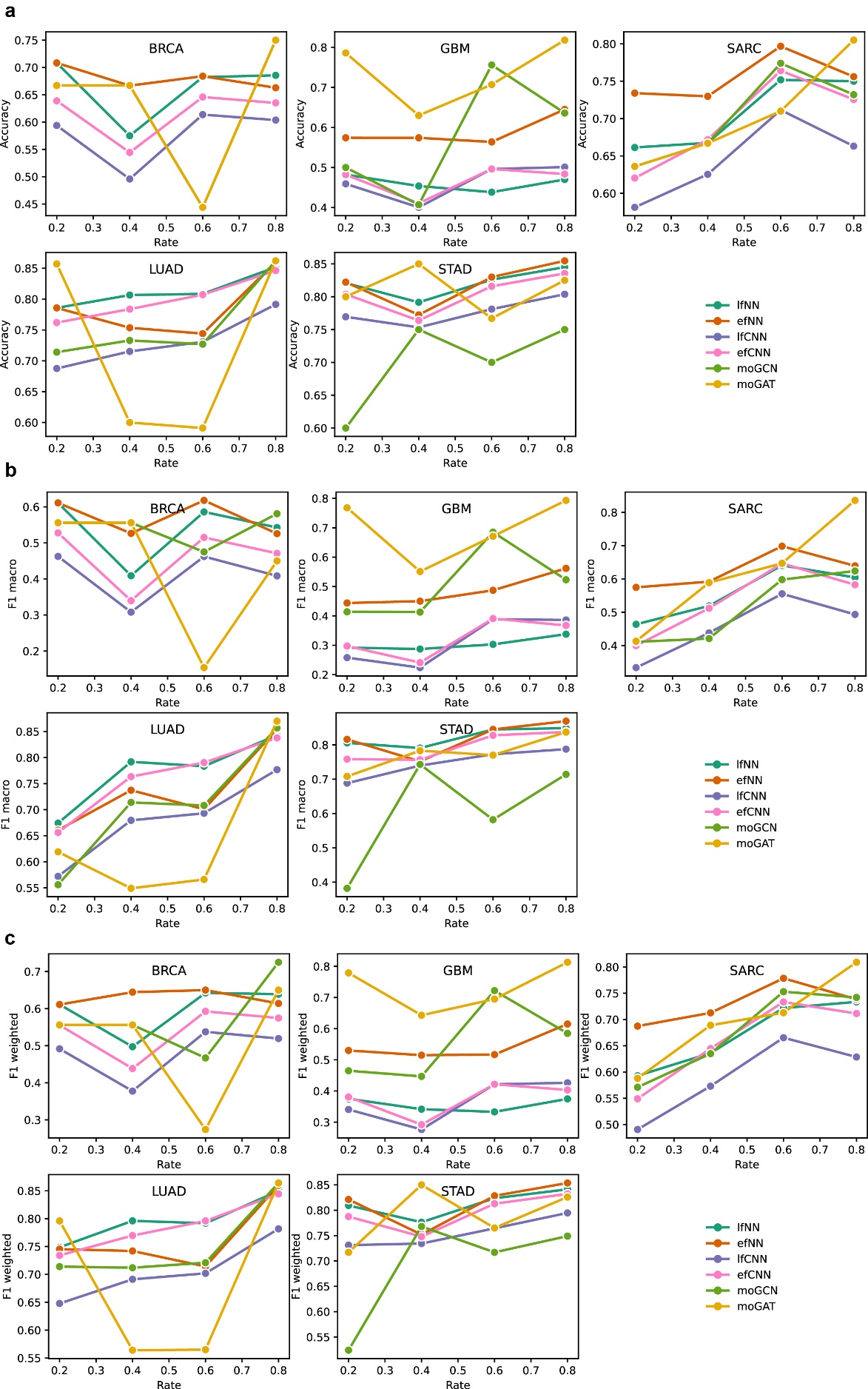


**Figure S1 Data reduction experiment on cancer benchmark datasets used in classification task.** Accuracy (**a**), F1 macro (**b**), F1 weighted (**c**) of the six unsupervised methods for classification under 20%, 40%, 60%, 80% of the total samples in the original data, respectively.
