# Supplementary material for: A benchmark study of deep learning-based multi-omics data fusion methods for cancer
Source: Genome Biol. 2022 Aug 9;23:171. doi: 10.1186/s13059-022-02739-2 (PMC9361561; doi:10.1186/s13059-022-02739-2)
Supplement: Supplementary file 2 — Additional file 2. Review History [file 13059_2022_2739_MOESM2_ESM.docx]

Review History

**First round of review**

**Reviewer 1**

**Were you able to assess all statistics in the manuscript, including the appropriateness of statistical tests used?**

Yes

**Comments to author:**

In this study, Leng et al. comprehensively evaluated 10 representative deep learning methods on a multi-omics dataset of simulations, single cells, and cancer. First, the authors compared the performance of different deep learning methods in searching for retrieving ground-truth sample classification and clustering of simulated multi-omics and single-cell multi-omics datasets. The authors then evaluated the capabilities of three end-to-end deep learning methods for breast cancer and GBM classification. Next, two different metrics were then used as internal indicators to evaluate the performance of the method in the clustering tasks. Finally, the authors compared the performance of the seven deep learning methods by identifying feature associations between survival and clinical annotation. The results showed that moGCN achieved the most promising classification performance; the lfNN also showed efficient performance across these complementary contexts.

Indeed, methods for analyzing multi-omics data on cancer are important, and comparing the performance of previously published methods may provide important information for researchers working in this area. On the other hand, I think there is a problem in terms of originality whether this manuscript is appropriate for publication in the high-impact journal Genome Biology in that it only presents the results of a comparison of existing methods. The issues are itemized below.

[Major issues]

1. There is an issue of originality; this study only compared existing methods and did not propose any new methods by the authors. In particular, deep learning methods are constantly evolving, and existing methods tend to quickly become old fashioned. Judging from this perspective, it is questionable to allow this manuscript to be published in the high-impact journal Genome Biology.

2. In general, deep learning methods tend to be black boxes. The authors also compared the performance of multiple deep learning methods, but I have the impression that the logical discussion is not properly conducted. For example, three models efNNA, lfNN, and moGCN were used for classification (efNN and lfNN are based on FCNN, which is a different data fusion method). Although the reason for their use in previous reports is given, there is a lack of discussion on whether FCNN and moGCN are appropriate for multi-omics analysis, and it is questionable to discuss their superiority after only testing a limited number of methods. It is also stated that moGCN obtained the highest ranked results in the classification problem, but the logical explanation is insufficient.

3. Table 4 shows that efNN performs better than moGCM in breast cancer in terms of accuracy and F1 weighted. This may suggest that the best model depends on the type of cancer and the type of data, but there is no in-depth discussion of this point in this manuscript and it should be considered carefully.

4. If clinical application is important, I believe that research that does not rely solely on databases is necessary, such as verification using data that the authors have actually collected prospectively. Limitations of the models used to date should also be discussed, and the techniques and methodologies needed to improve them in the future should be described.

**Reviewer 2**

**Were you able to assess all statistics in the manuscript, including the appropriateness of statistical tests used?**

Yes

**Comments to author:**

Leng et al. presented a large-scale benchmarking analysis for several deep learning-based multi-omics integration methods. The authors have evaluated a variety of tasks with several metrics for benchmarking. This study is timely and very useful for the community, since many new tools have been published yet not much work has been done to benchmark their performance. However, there are several key technical limitations. Most importantly, the descriptions of datasets and experiment setup are unclear, making reproducibility a concerning issue. Some analyses are not appropriate for evaluating the methods.

Major points:

1. Why is Python a selection criterion? R packages can also be worth reviewing? The authors should clarify the selection criteria.

2. Why the original method names were not used? The author named the methods after their underlying deep learning architecture. However, it is not appropriate to just use one method implementation to represent the underlying architecture. For example, MOGONET is one implementation of moGCN and other implementations that are based on moGCN might be potentially better.

3. The authors briefly described the ten methods. However, the descriptions were not consistent. For example, what omics were included for the original usage of the tools? This was described for AE but not others.

4. The experiment using the simulated dataset was unclear. What was being predicted? How many data points were generated? "The simulated multi-omics data were fused by our DL-based evaluation methods…to perform the classification tasks", does this mean that the seven were used for dimensionality reduction, and then the three for classification? If this is the case, then there would be 21 combinations for the final classification, because the second step was dependent on the first step?

5. The authors used four metrics to evaluate the clustering performance. How consistent was the performance across different metrics? How should one interpret the results? It's not straightforward to see if any method outperforms the others across all metrics.

6. Similar to #4, the experiment for the single-cell data was also unclear. What was it classifying? Which dimensionality method was used prior to the classification?

7. Similar to #5, the final conclusion was not clear for the single-cell data benchmarking. The authors claimed "Overall, lfAE, lfDAE, and efVAE obtained the most promising clustering results among the seven DL methods.", which couldn't be supported by any figure.

8. For the TCGA dataset benchmarking, how many samples were included for BRCA and GBM?

9. For the clustering benchmarking using TCGA data, were the methods running with 2-6 clusters for each cancer dataset independently? If so, what label was used to calculate the Silhouette and Davies Bouldin score within each cancer type?

10. The analysis and interpretation of Fig4c and 4d might be inappropriate. The authors first performed dimensionality reduction to reduce the features into ten and then associated these ten features with clinical outcomes and annotations. However, the number of significant associations from the ten features did not reflect any kind of performance. The ten features in the latent space are likely to be orthogonal. Having all ten features significantly associated with any one specific clinical feature means all ten features captured similar information, which may indicate an inferior performance of the dimensionality reduction.

11. Fig5 should quantitatively summarise the evaluations, not qualitatively as in the current form.

12. For each of the three experiments, how was the evaluation conducted? How much data were used in the test set? Was cross-validation used?

Minor points:

1. In Table 1, row 1 and 3 are identical?

2. The authors included computational details for each benchmarked method, but these details can be referred to the original publication. These details might be necessary.

3. "To ensure the authenticity of the assessment, the data we used are all from benchmark cancer datasets", does the referenced website has the up-to-date data, as the data are not directly downloaded from TCGA? Also BRCA is not listed on that page. What is the source of the breast cancer data?

**Authors Response**

**Point-by-point responses to the reviewers’ comments:**

**Referee #1:**

**Major issues:**

Comment 1:

There is an issue of originality; this study only compared existing methods and did not propose any new methods by the authors. In particular, deep learning methods are constantly evolving, and existing methods tend to quickly become old fashioned. Judging from this perspective, it is questionable to allow this manuscript to be published in the high-impact journal Genome Biology.

*Response：*

*Thank you for this constructive and important advice.*

*We are sorry for the unclear description of the research motivation in previous manuscript. We think that the main contribution of this research is to lay the foundation for future multi-omics analysis. Currently, many new deep learning (DL)-based multi-omics data fusion methods have been proposed and they showed promising performance in the multi-omics fusion. However, there are few works to evaluate these methods. Therefore, we comprehensively evaluated 16 DL-based methods that are representative of multi-omics fusion methods in three different contexts, i.e., simulated multi-omics datasets, single-cell multi-omics datasets, and cancer multi-omics datasets. And these methods cover the mainstream DL-based methods that are currently applied to multi-omics data fusion.*

*According to your suggestion and reminder, we have re-organized the manuscript in three main ways to response to your comment.*

***(1) Re-organize the results.***

*We added more details on how the evaluation and demonstration was performed and added several schematic diagrams to make the evaluation process clearer. Meanwhile, we described the motivation of each sub-benchmark (i.e., simulated multi-omics datasets, single-cell multi-omics datasets, and cancer multi-omics datasets). Moreover, in order to show the performance of different methods on different datasets more comprehensively and clearly, we introduced different tasks and various evaluation indices for evaluation, and re-organized the results and conclusions of each sub- benchmark.*

***(2) Re-write the Discussion.***

*We summarized the conclusions of each sub-benchmark. Then, to make our evaluation more objective, we defined a “unified score” to unify the results of each indicator as the final comprehensive evaluation indicator. Based on the benchmarking results, we provide suggestions for biologists to choose appropriate methods in different scenarios and added more logical explanations for the best performing methods. After that, we discussed the limitation in this benchmark study. More importantly, we give guidelines of algorithm improvement for researches focusing on algorithm design of multi-omics data fusion.*

***(3) Design and compare three original methods for data fusion.***

*According to your suggestion, we designed three original methods (i.e., efVAE, efSVAE, efmmdVAE) that are not published in previous researches and added them into this benchmark study.*

*Overall, this benchmark study not only provides a reference for biomedical researchers to choose appropriate DL-based multi-omics data fusion methods for their purpose, but also suggests the future directions for the development of more effective multi-omics data fusion methods. We think that this benchmark study could be very useful for the progress of future multi-omics analysis.*

Comment 2:

In general, deep learning methods tend to be black boxes. The authors also compared the performance of multiple deep learning methods, but I have the impression that the logical discussion is not properly conducted. For example, three models efNNA, lfNN, and moGCN were used for classification (efNN and lfNN are based on FCNN, which is a different data fusion method). Although the reason for their use in previous reports is given, there is a lack of discussion on whether FCNN and moGCN are appropriate for multi-omics analysis, and it is questionable to discuss their superiority after only testing a limited number of methods. It is also stated that moGCN obtained the highest ranked results in the classification problem, but the logical explanation is insufficient.

*Response：*

*Thank you for this important advice.*

*According to your suggestions, we added more logical explanations for the best performing methods and provided suggestions for researchers to choose appropriate DL-based multi-omics data fusion methods in different scenarios in the third paragraph of “Discussion and Conclusion”. The revisions are marked in blue color in the revised manuscript. Below we give detailed explains on whether the selected methods are appropriate for multi-omics analysis.*

*First, the DL-based methods we selected are designed for multi-omics fusion in the original researches, but they were used on different input omics data and different downstream tasks. We defined two rules for the methods selection: (1) The original models of the selected methods have open-source code; and (2) The original models used multi-omics fusion and the data fusion part can be extracted separately. To evaluate which method is more appropriate for multi-omics analysis and make different methods comparable, in this benchmark, we first extracted the data fusion part from the original model and then compare the performance on unified datasets and tasks. Furthermore, to evaluate the performance of the methods in different scenarios, we used three types of multi-omics datasets: simulated data, single-cell data, and cancer data.*

*Second, based on the benchmark results, we can provide recommendations for the use of each method in different scenarios. And according to your advice, we added more logical explanations to guide the selection and improvement of methods in the revised manuscript.*

*Furthermore, in order to better discuss the methods’ superiority, we added more DL-based methods. For NN-based methods, we added efCNN and lfCNN; for GNN-based methods, we added moGAT; for AE-based methods, we designed three original methods: efVAE, efSVAE, and efmmdVAE. The increase of methods is benefit for comprehensive evaluation and more reliable conclusions.*

Comment 3:

Table 4 shows that efNN performs better than moGCM in breast cancer in terms of accuracy and F1 weighted. This may suggest that the best model depends on the type of cancer and the type of data, but there is no in-depth discussion of this point in this manuscript and it should be considered carefully.

*Response：*

*Thank you for this constructive advice to improve our manuscript.*

*In cancer datasets, to better explore how the cancer type influences the benchmarking results, we added three additional cancer datasets with different data size and subtyping labels to our classification task datasets. At last, we have five different cancer data sets with ground-truth cancer subtypes from TCGA: breast cancer (BRCA), glioblastoma (GBM), sarcoma (SARC), lung adenocarcinoma (LUAD) and stomach cancer (STAD). Notably, other cancer datasets in TCGA have no ground-truth cancer subtypes labels, so we did not include them in the benchmark of classification tasks. We further investigated how the cancer type and data size influence the benchmarking results. And We have added the corresponding parts in the fourth and fifth paragraphs in “Evaluation of deep learning-based multi-omics data fusion methods on cancer datasets” section of Results in current manuscript.*

(1) ***The description of the datasets.***

*We obtained 59, 272, 206, 144, and 198 samples for BRCA, GBM, SARC, LUAD, and STAD, respectively. The subtypes for each cancer were listed in RTable 1 (i.e., Table 4 in current manuscript).*

***RTable 1.*** *The sizes and features of cancer benchmark datasets used in classification task.*

| *Cancers* | *Categories (cancer subtypes)* | *# of samples* | *# of features* |
| --- | --- | --- | --- |
|  |  |  | *Exp, Meth, miRNA* |
| *BRCA* | *Luminal A: 28, Luminal B: 15, Basal-like: 12, HER2-enriched: 4* | *59* | *6000, 5000, 892* |
| *GBM* | *Proneural: 71, Classical: 70, Mesenchymal: 84, Neural: 47* | *272* | *6000, 5000, 534* |
| *SARC* | *DDLPS: 50, LMS: 80, UPS: 44, MFS: 17, MPNST: 5, SS: 10* | *206* | *6000, 5000, 1046* |
| *LUAD* | *TRU: 51, PI: 52, PP: 41* | *144* | *6000, 5000, 554* |
| *STAD* | *EBV: 20, MSI: 38, GS: 43, CIN: 97* | *198* | *6000, 5000, 519* |

*#, number; Exp, gene expression; Meth, DNA methylation; miRNA, miRNA expression; DDLPS, dedifferentiated liposarcoma; LMS, leiomyosarcoma; UPS, undifferentiated pleomorphic sarcoma; MFS, myxofibrosarcoma; MPNST, malignant peripheral nerve sheath tumor; SS, synovial sarcoma; TRU, formerly bronchioid; PI, formerly squamoid; PP, formerly magnoid; EBV, Epstein–Barr virus; MSI, microsatellite instability; GS, genomically stable; CIN, chromosomal instability.*

(2) ***The exploration of how cancer type affects performance.***

*Based on the new benchmarking results (RTable 2, i.e., Table 5 in current manuscript), it can be seen that all methods do not perform well on BRCA. This is because BRCA has significantly fewer samples than other cancers. For GBM, although it has the largest number of samples among the five cancers, most methods do not achieve good performance. According to investigation, we found that the subtype labels of GBM may have some deviations. Recent researches suggested that GBM should be classified into three subtypes instead of the four* subtypes labeled by TCGA [1-4].

**RTable 2.** Performance of six supervised methods on cancer benchmark datasets used in classification task

| Methods |  | BRCA | |  |  | GBM |  |  | SARC |  |  | LUAD |  |  | STAD |  |
| --- | --- | --- | --- | --- | --- | --- | --- | --- | --- | --- | --- | --- | --- | --- | --- | --- |
|  | Accuracy | | F1 macro | F1 weighted | Accuracy | F1 macro | F1 weighted | Accuracy | F1 macro | F1 weighted | Accuracy | F1 macro | F1 weighted | Accuracy | F1 macro | F1 weighted |
| lfNN | 0.475 | | 0.161 | 0.306 | 0.309 | 0.118 | 0.146 | 0.767 | 0.544 | 0.739 | **0.896** | **0.892** | **0.894** | 0.843 | 0.853 | 0.844 |
| efNN | 0.660 | | 0.544 | 0.617 | 0.588 | 0.512 | 0.554 | 0.772 | 0.668 | 0.771 | 0.861 | 0.848 | 0.855 | **0.868** | **0.876** | **0.866** |
| lfCNN | 0.525 | | 0.248 | 0.382 | 0.548 | 0.435 | 0.481 | 0.587 | 0.328 | 0.525 | 0.688 | 0.683 | 0.683 | 0.657 | 0.543 | 0.606 |
| efCNN | 0.594 | | 0.431 | 0.546 | 0.455 | 0.347 | 0.372 | 0.786 | 0.626 | 0.768 | 0.868 | 0.866 | 0.868 | 0.818 | 0.834 | 0.818 |
| moGCN | 0.600 | | **0.589** | 0.601 | 0.647 | 0.633 | 0.585 | **0.846** | **0.852** | **0.851** | 0.861 | 0.860 | 0.861 | 0.740 | 0.733 | 0.716 |
| moGAT | **0.667** | | 0.545 | **0.671** | **0.721** | **0.702** | **0.73** | 0.731 | 0.719 | 0.735 | 0.861 | 0.861 | 0.863 | 0.820 | 0.779 | 0.819 |

(3) ***The exploration of how data size affects performance.***

*In order to further explore how the data size influence the benchmarks, we reduced the amount of data and observed the effects of data reduction. Specifically, we randomly selected 20%, 40%, 60%, 80% of the total samples in the original data. Then we evaluated all six methods under different amounts of data. The results of the data reduction experiment are shown in RFig. 1 (i.e., Figure S1 in Additional File 1). Except for two GNN-based methods (moGAT and moGCN), the performances of other methods are impaired when the amount of data decreases. The performance of the two GNN-based methods fluctuated greatly. This may be because that the network structure changes greatly with the change of data for the GNN-based methods.*

***RFig. 1 Data reduction experiment on cancer benchmark datasets used in classification task.*** *Accuracy (****a****), F1 macro (****b****), F1 weighted (****c****) of the six unsupervised methods for classification under 20%, 40%, 60%, 80% of the total samples in the original data, respectively.*

Comment 4:

If clinical application is important, I believe that research that does not rely solely on databases is necessary, such as verification using data that the authors have actually collected prospectively. Limitations of the models used to date should also be discussed, and the techniques and methodologies needed to improve them in the future should be described.

*Response：*

*Thanks for this suggestion.*

*In the evaluation of deep learning-based multi-omics data fusion methods on cancer datasets, we used the Cancer Genome Atlas (TCGA) data. The Cancer Genome Atlas (TCGA) has accumulated comprehensive multi-omics molecular profiles of 33 cancer types from more than 10,000 patients (e.g., genomics, transcriptomics, proteomics, and epigenomics) [5]. The TCGA data is very representative and is widely used in various benchmark studies and biomedical researches [6-15]. At present, there have been a lot of achievements in the research of biomedical based on TCGA. The TCGA data is significantly important for guidance prevention, diagnosis, and treatment of various cancers. Therefore, the benchmarking results obtained from TCGA data could be extended to other clinical cancer data.*

*Moreover, using authoritative data that has been verified by many studies is beneficial for other researchers to reproduce our results. The unified evaluation data is also beneficial for subsequent algorithm researchers to develop and evaluate algorithms on the same basis.*

*In the future, based on this benchmarking results, we will design better algorithms and apply them to first-hand clinical data, so as to better guide clinical practice.*

*Furthermore, according to your suggestions, we discussed techniques and methodologies needed to improve existing methods and suggested the future directions for the development of more effective multi-omics data fusion methods. We have added the corresponding parts in the last paragraph in “Discussion and Conclusion” section in current manuscript. Below we give discussions on techniques and methodologies needed to improve existing methods.*

*In spite of the great progress in multi-omics data fusion brought by the existing DL methods, there is still room for future improvement from a computational perspective.*

***(1) Dealing with class imbalance.*** *As demonstrated in the evaluation on simulated datasets, compared with the condition that clusters have variable random sizes, most methods perform better in the condition that all clusters have the same size. Imbalanced classes could impair model performance. Further extensions of DL-based multi-omics data fusion methods could try to cope with class imbalance problem by applying cost-sensitive learning [16, 17], ensemble learning (e.g., bagging and boosting) and so on.*

***(2) Combining AE and GNN.*** *Based on the evaluation results, we observed that GNN-based methods achieve good performance. Graph auto-encoder (GAE), which combines AE and GNN, has achieved success on many tasks [18-20]. Applying GAE-based methods on cancer and single-cell multi-omics data fusion could be promising and is worthy of further exploration.*

***(3) Designing algorithms that accommodate the missing observations.*** *Multi-omics data fusion is often accompanied by the absence of samples in one or several omics. Taking more omics types into account and using the sample sets consisting of all omics data types can lead to limited sample size. One solution is to infer the missing features. Based on the observation that different omics are not completely independent and can be correlated, we can infer the missing features by using the complementary information of different omics. For example, in the field of chemoinformatics, Martino et al. designed a model by employing siamese neural network to massively infer the missing features for the ~800,000 molecules [21]. In addition to missing features inference, using generative adversarial networks (GAN) to generate data similar to a real dataset is also a promising method. Because GAN-based algorithms are capable of learning and imitating any distribution of data, Ahmed et al. [22] employed GAN to fuse two omics data. But this GAN-based model can only be applied to specific types of data with explicit interactions (e.g., miRNA-mRNA interaction). Although GAN-based multi-omics data fusion algorithms have some limitations currently, they deserve to be further explored in the application of missing value imputation [23].*

***(4) Developing explainable DL methods.*** *Most of the state-of-the-art DL methods lack interpretation, which is an increasing demand in biomedical field. For the “black-box” of DL models, it is difficult to elucidate the underlying biological mechanisms. One emerging approach is to embed prior biological knowledge into the DL models. There are several studies that use knowledge-embedded algorithms to provide explanations [24-26]. Mao et al. [24] performed dimensionality reduction on high-dimensional single-cell transcriptome data and provided explanations by embedding prior knowledge into matrix factorization approach. Gut et al. [25] proposed a knowledge-embedded VAE by restricting the structure of VAE to mirror gene-pathway memberships and applied the model on the dimensionality reduction of single cell RNA-seq data. Similarly, developing knowledge-embedded DL methods for multi-omics data fusion is promising and can provide new insight for the underlying mechanisms. Overall, based on this comprehensive benchmark study and several potential improvement strategies, developers could make more progress on multi-omics data fusion.*

***Reference***

*1.	Mao XG, Xue XY, Wang L, Lin W, Zhang X. Deep learning identified glioblastoma subtypes based on internal genomic expression ranks. BMC Cancer. 2022;22(1):86.*

*2.	Wang Q, Hu B, Hu X, Kim H, Squatrito M, Scarpace L, et al. Tumor Evolution of Glioma-Intrinsic Gene Expression Subtypes Associates with Immunological Changes in the Microenvironment. Cancer Cell. 2017;32(1):42-56 e6.*

*3.	Hu B, Ruan Y, Wei F, Qin G. Identification of three glioblastoma subtypes and a six-gene prognostic risk index based on the expression of growth factors and cytokines. Am J Transl Res. 2020;12(8):4669-82.*

*4.	Zhang P, Xia Q, Liu L, Li S, Dong L. Current Opinion on Molecular Characterization for GBM Classification in Guiding Clinical Diagnosis, Prognosis, and Therapy. Front Mol Biosci. 2020;7:562798.*

*5.	Tomczak K, Czerwinska P, Wiznerowicz M. The Cancer Genome Atlas (TCGA): an immeasurable source of knowledge. Contemp Oncol (Pozn). 2015;19(1A):A68-77.*

*6.	Cantini L, Zakeri P, Hernandez C, Naldi A, Thieffry D, Remy E, et al. Benchmarking joint multi-omics dimensionality reduction approaches for the study of cancer. Nat Commun. 2021;12(1):124.*

*7.	Franco EF, Rana P, Cruz A, Calderón VV, Azevedo V, Ramos RTJ, et al. Performance Comparison of Deep Learning Autoencoders for Cancer Subtype Detection Using Multi-Omics Data. Cancers. 2021;13(9):2013.*

*8.	Pierre-Jean M, Deleuze JF, Le Floch E, Mauger F. Clustering and variable selection evaluation of 13 unsupervised methods for multi-omics data integration. Brief Bioinform. 2020;21(6):2011-30.*

*9.	Herrmann M, Probst P, Hornung R, Jurinovic V, Boulesteix AL. Large-scale benchmark study of survival prediction methods using multi-omics data. Brief Bioinform. 2021;22(3).*

*10.	Rappoport N, Shamir R. Multi-omic and multi-view clustering algorithms: review and cancer benchmark. Nucleic Acids Res. 2018;46(20):10546-62.*

*11.	Tini G, Marchetti L, Priami C, Scott-Boyer MP. Multi-omics integration-a comparison of unsupervised clustering methodologies. Brief Bioinform. 2019;20(4):1269-79.*

*12.	Berger AC, Korkut A, Kanchi RS, Hegde AM, Lenoir W, Liu W, et al. A Comprehensive Pan-Cancer Molecular Study of Gynecologic and Breast Cancers. Cancer Cell. 2018;33(4):690-705 e9.*

*13.	Cancer Genome Atlas Research Network. Electronic address aadhe, Cancer Genome Atlas Research N. Integrated Genomic Characterization of Pancreatic Ductal Adenocarcinoma. Cancer Cell. 2017;32(2):185-203 e13.*

*14.	Hoadley KA, Yau C, Wolf DM, Cherniack AD, Tamborero D, Ng S, et al. Multiplatform analysis of 12 cancer types reveals molecular classification within and across tissues of origin. Cell. 2014;158(4):929-44.*

*15.	Liu J, Lichtenberg T, Hoadley KA, Poisson LM, Lazar AJ, Cherniack AD, et al. An Integrated TCGA Pan-Cancer Clinical Data Resource to Drive High-Quality Survival Outcome Analytics. Cell. 2018;173(2):400-16 e11.*

*16.	Zhi-Hua Z, Senior M, IEEE, Xu-Ying L. Training cost-sensitive neural networks with methods addressing the class imbalance problem. IEEE TRANSACTIONS ON KNOWLEDGE AND DATA ENGINEERING. 2006;18(1):63-77.*

*17.	Khan SH, Hayat M, Bennamoun M, Sohel FA, Togneri R. Cost-Sensitive Learning of Deep Feature Representations From Imbalanced Data. IEEE Trans Neural Netw Learn Syst. 2018;29(8):3573-87.*

*18.	Yang F, Fan K, Song D, Lin H. Graph-based prediction of Protein-protein interactions with attributed signed graph embedding. BMC Bioinformatics. 2020;21(1):323.*

*19.	Karimi M, Hasanzadeh A, Shen Y. Network-principled deep generative models for designing drug combinations as graph sets. Bioinformatics. 2020;36(Suppl_1):i445-i54.*

*20.	Li H, Sun Y, Hong H, Huang X, Tao H, Huang Q, et al. Inferring transcription factor regulatory networks from single-cell ATAC-seq data based on graph neural networks. Nature Machine Intelligence. 2022;4(4):389-400.*

*21.	Bertoni M, Duran-Frigola M, Badia IMP, Pauls E, Orozco-Ruiz M, Guitart-Pla O, et al. Bioactivity descriptors for uncharacterized chemical compounds. Nat Commun. 2021;12(1):3932.*

*22.	Ahmed KT, Sun J, Yong J, Zhang W. Multi-omics data integration by generative adversarial network. Bioinformatics. 2022;38(1):179-86.*

*23.	Xu Y, Zhang Z, You L, Liu J, Fan Z, Zhou X. scIGANs: single-cell RNA-seq imputation using generative adversarial networks. Nucleic Acids Res. 2020;48(15):e85.*

*24.	Mao W, Zaslavsky E, Hartmann BM, Sealfon SC, Chikina M. Pathway-level information extractor (PLIER) for gene expression data. Nat Methods. 2019;16(7):607-10.*

*25.	Gut G, Stark SG, Rätsch G, Davidson NR. pmVAE: Learning Interpretable Single-Cell Representations with Pathway Modules. bioRxiv. 2021.*

*26.	Rybakov S, Lotfollahi M, Theis FJ, Wolf FA. Learning interpretable latent autoencoder representations with annotations of feature sets. bioRxiv. 2020.*

**Referee #2:**

**General comment:**

Leng et al. presented a large-scale benchmarking analysis for several deep learning-based multi-omics integration methods. The authors have evaluated a variety of tasks with several metrics for benchmarking. This study is timely and very useful for the community, since many new tools have been published yet not much work has been done to benchmark their performance. However, there are several key technical limitations. Most importantly, the descriptions of datasets and experiment setup are unclear, making reproducibility a concerning issue. Some analyses are not appropriate for evaluating the methods.

*Response：*

*Thank you for your positive comments.*

*For your concerns about technical limitations, we have made corresponding modifications in the revised manuscript and answered your questions in the following point-by-point response.*

**Major points:**

Comment 1:

Why is Python a selection criterion? R packages can also be worth reviewing? The authors should clarify the selection criteria.

*Response:*

*We are very sorry for our inappropriate expression in the manuscript, and we have corrected this inappropriate expression in the revised manuscript. We have changed the criteria with the following two rules as our corrected methods selection criteria: (1) The original models of the selected methods have open-source code; and (2) The original model used multi-omics data and the data fusion part can be extracted separately, so that we can evaluate the data fusion on unified datasets and tasks.*

*According to the methods selection criteria, all of the methods we collected were implemented in Python. This may be due to the fact that Python is more comprehensive than R in deep learning. Although R can interface to TensorFlow and Keras to conduct deep learning, it is not the mainstream in deep learning. Compared with R, Python has more deep learning libraries, frameworks, and communities. In the future, with the introduction of more deep learning libraries on R, more deep learning frameworks implemented by R will be added to the benchmark study.*

Comment 2:

Why the original method names were not used? The author named the methods after their underlying deep learning architecture. However, it is not appropriate to just use one method implementation to represent the underlying architecture. For example, MOGONET is one implementation of moGCN and other implementations that are based on moGCN might be potentially better.

*Response:*

*Thank you for this constructive and important advice.*

***(1)	The reasons of not using the original method names.***

*In this benchmark study, we investigated DL-based multi-omics data fusion methods had been published and evaluated the data fusion framework of original method based on the open-source code. Most of these models in previous publications were used with different strategies (early or late fusion) to fuse data first, and then the integrated data were applied in various downstream tasks. Early fusion means each omics data are fused first and then inputted into DL-based models. Late fusion means the multi-omics data are inputted into DL-based models first and then fused for downstream tasks. Due to the different input omics data and different downstream tasks, it is difficult to compare these methods directly.*

*To make different methods comparable, in this study, we first extract the data fusion part from the original model and then compare the performance on unified datasets and tasks. In addition, the original methods are usually named according to the downstream tasks. Using the original method names on the unified benchmark datasets and tasks could be confusing.*

*Given all that, we renamed the methods according to their structural characteristics in order to help readers understand the essential differences between the methods.*

***(2) Discussion on different implementations of the same data fusion framework***

*In original publications, different method implementations in the same data fusion framework were used to perform different tasks (e.g., cancer subtyping and clustering of single-cell multi-omics data) based on different multi-omics data (e.g., cancer data and single-cell data). These various implementations cannot be directly compared. To* *imitate the different implementations of the same data fusion framework, we extract the data fusion part from the original model and designed three different scenarios: simulated data, single-cell data, and cancer data. The implementations of the same data fusion framework in three different kinds of datasets represent different implementations of the same framework. The comprehensive evaluation of the three implementations could reflect the effectiveness and generalizability of the corresponding data fusion frameworks. And based on the benchmarking results, we can provide recommendations for the use of each data fusion framework in different scenarios and suggest the future directions for the development of more effective multi-omics data fusion methods.*

***(3)	For completeness of the evaluation, more DL-based methods are added.***

*In our manuscript, there are three typical deep learning architectures, i.e., AE, NN and GNN, and we have evaluated multiple methods under each architecture. Inspired by your suggestions about this question, in order to more comprehensively evaluate different architectures, we not only considered three different scenarios, but also added some new methods under each architecture. For NN-based methods, we added efCNN and lfCNN; for GNN-based methods, we added moGAT; for AE-based methods, we designed three original methods: efVAE, efSVAE, and efmmdVAE. The increase of methods is benefit for comprehensive evaluation and more reliable conclusions.*

*Comment 3:*

*The authors briefly described the ten methods. However, the descriptions were not consistent. For example, what omics were included for the original usage of the tools? This was described for AE but not others.*

*Response:*

*Thank you for this detailed comment. We have re-described these methods briefly in the current manuscript.*

*In order to provide a clear presentation of the evaluation methods, we have added a table of the descriptions of the evaluated DL-based multi-omics fusion methods (RTable.1). The table contains methods, feature types, applications, and reference.*

***RTable 1.*** *Descriptions of the evaluated DL-based multi-omics fusion methods*

| *Methods* | *Feature types* | *Applications* | *Reference* |
| --- | --- | --- | --- |
| *lfNN* | *Gene expression and the drug pairs’ chemical structure* | *Synergistic drug combination prediction* | *Kuru et al. [1]* |
| *efNN* | *Gene expression and the drug pairs’ chemical structure* | *Synergistic drug combination prediction* | *Preuer et al. [2]* |
| *lfCNN* | *CNV, gene expression and clinical data* | *Cancer subtypes classification* | *Islam et al. [3]* |
| *efCNN* | *Variation counts, gene expression, QTANs/QTALs number, and WGCNA module features* | *Gene regulation mechanisms prediction* | *Fu et al. [4]* |
| *moGCN* | *Gene expression, DNA methylation and miRNA expression* | *Prognosis and biomarker identification* | *Wang et al. [5]* |
| *moGAT* | *mRNA expression and proteomic data* | *Disease diagnosis and prognosis* | *Xing et al. [6]* |
| *lfAE* | *Gene expression, miRNA expression, and DNA methylation* | *Clinical target variables prediction* | *Ma et al. [7]* |
| *efAE* | *Gene expression, miRNA expression, DNA methylation and CNV* | *Prognosis* | *Lee et al. [8]* |
| *lfDAE* | *Gene expression, miRNA expression and DNA methylation* | *Cancer subtypes classification* | *Poirion et al. [9]* |
| *efDAE* | *Gene expression, miRNA expression and CNV* | *Cancer subtypes classification* | *Guo et al. [10]* |
| *lfVAE* | *DNA methylation and gene expression* | *Survival and clinical analysis* | *Our original method in this work* |
| *efVAE* | *Gene expression, miRNA expression and DNA methylation* | *Cancer subtypes classification* | *Zhang et al. [11]* |
| *lfSVAE* | *Gene expression, point mutations, and CNV* | *Survival and clinical analysis* | *Our original method in this work* |
| *efSVAE* | *Gene expression, miRNA expression and DNA methylation* | *Cancer subtypes classification* | *Ronen et al. [12]* |
| *lfmmdVAE* | *DNA methylation, gene expression and CNV* | *Survival and clinical analysis* | *Our original method in this work* |
| *efmmdVAE* | *Gene expression, miRNA expression and DNA methylation* | *Patient stratification* | *Hira et al. [13]* |

*Comment 4:*

*The experiment using the simulated dataset was unclear. What was being predicted? How many data points were generated? "The simulated multi-omics data were fused by our DL-based evaluation methods…to perform the classification tasks", does this mean that the seven were used for dimensionality reduction, and then the three for classification? If this is the case, then there would be 21 combinations for the final classification, because the second step was dependent on the first step?*

*Response:*

*We are very sorry for our obscure expression in the manuscript. We have re-described this experiment in the current manuscript. To facilitate understanding, we have added a schematic diagram showing how the methods were evaluated on the simulated multi-omics datasets (RFig. 1, i.e., Fig. 2 in current manuscript)*

***RFig. 1 Workflow of the evaluation on simulated multi-omics datasets.*** ***a*** *InterSIM CRAN package generated three kinds of omics data that were used as input.* ***b*** *Supervised DL methods are evaluated in the classification tasks. The performance of these methods was based on 4-fold cross validation and was evaluated by three metrics: accuracy, F1 macro, and F1 weighted score.* ***c*** *Unsupervised DL methods are applied to fuse the simulated multi-omics data to obtain 5-dimension, 10-dimension, and 15-dimension embeddings first. Then k-means algorithm is used to cluster the multi-omics dimensionality reduction results. We employed Jaccard Index, C-index, silhouette score and Davies Bouldin score as the evaluation indexes of clustering.*

*The simulated multi-omics datasets were generated using the InterSIM CRAN package [14]. This package can generate complex and interrelated multi-omics data, including DNA methylation, mRNA gene expression, and protein expression data. We generated 100 simulated samples with 1000 dimensional features. In generation process, the cluster number parameter of 100 simulated samples is set to 5, 10, and 15. Furthermore, we generated each cluster of samples in two conditions, either all clusters have the same size, or the clusters have variable random size. It simulates a real application scenario that the proportion of samples belonging to each cluster (subtype) could be the same or different.*

*Six supervised DL methods are evaluated in the classification tasks. These six supervised methods are intrinsically designed for sample classification. Their performances were then compared based on the classification results.*

*For the ten other unsupervised DL methods, they are applied to fuse the simulated multi-omics data to obtain 5-dimension, 10-dimension, and 15-dimension embeddings first. The dimension of embeddings is set according to number of clusters in the simulated multi-omics data. Then we used k-means algorithm to cluster the multi-omics dimensionality reduction results. The clustering of the samples is finally obtained to compare the performances of the ten unsupervised methods.*

*To quantitatively evaluate the performance of the supervised DL methods, we partitioned the dataset into training and test sets with the ratio of 3:1, and performed 4-fold cross-validation. Three metrics (accuracy, F1 macro, and F1 weighted score) are calculated (see Methods).*

*For the clustering tasks, four metrics (i.e., Jaccard Index, C-index, silhouette score, and Davies Bouldin score) are employed to evaluate the performance.*

Comment 5:

The authors used four metrics to evaluate the clustering performance. How consistent was the performance across different metrics? How should one interpret the results? It's not straightforward to see if any method outperforms the others across all metrics.

*Response：*

*Thank you for the constructive suggestion to improve our manuscript.*

*We employed various evaluation indices in this benchmark and the different indices describe the performance of the methods from different perspectives. For example, the external Jaccard Index was used to measure the agreement between multi-omics data fusion-based clusters and the ground-truth clusters, while the internal index silhouette score indicates how similar an object is to its own cluster (cohesion) compared to other clusters (separation).*

*We originally wanted to show the performance from multiple perspectives. However, inspired by your comment, we found that we indeed lacked a comprehensive performance evaluation index. Therefore, to make our evaluation more* *objective, we defined “unified score” to unify the results of each index as the final comprehensive evaluation index. The unified score is referring to the rank aggregation scheme for the Synapse Challenge (*[*https://doi.org/10.7303/syn6131484*](https://doi.org/10.7303/syn6131484)*). If one method was evaluated in more than one scenario (e.g., in simulated multi-omics dataset, all methods are evaluated on six scenarios: three cluster number × same size/random size), we used its average unified scores (RFig. 2, i.e., Fig. 9 in current manuscript). The definition of “unified score” was added in the section “Evaluation Metrics” in Methods in revised manuscript. We have added the benchmarking results based on the “unified score” in the third paragraph in “Discussion and Conclusion” section in revised manuscript.*

***RFig. 2 DL-based multi-omics data fusion methods benchmarked by average unified score in this study.*** ***a*** *The unified performances of supervised models in three different datasets.* ***c*** *The unified performances of unsupervised models in three different datasets. We used the highest unified score of every scenario as reference (marked 100%) to calculate the percentage.*

*Based on the results, for the classification tasks, moGAT ranked first on the three different multi-omics datasets (RFig. 2, i.e., Fig. 9 in current manuscript). For the clustering tasks, efVAE, lfmmdVAE, and lfAE were the top three methods on simulated datasets. lfAE, lfDAE and efmmdVAE were the top three methods on single-cell datasets. efmmdVAE, lfmmdVAE and efVAE were the top three methods on cancer datasets. Overall, GNN-based methods should be prioritized by researchers focusing on classification tasks. GNN-based methods structure the multi-omics data into similarity networks. The correlations among samples can be captured by the similarity networks. Therefore, the omics features and the geometrical structures of the data can be effectively utilized and the classification performance can benefit from it. When focusing on the clustering tasks, efmmdVAE, efVAE and lfmmdVAE should be prioritized. They have the most effective and consistent behavior across all the different benchmarks. These methods learn the probability distribution of the data. They have a layer of data means and standard deviations, which are used to generate new data. This allows for better generalization and flexibility of the learned embeddings. They can thereby be valuable tools for researchers interested in applying DL-based multi-omics data fusion methods with various biomedical problems.*

*Comment 6:*

*Similar to #4, the experiment for the single-cell data was also unclear. What was it classifying? Which dimensionality method was used prior to the classification?*

*Response：*

*We are sorry for our obscure expression in the manuscript. We have re-described this experiment in the current manuscript. To facilitate understanding, we have added a schematic diagram showing how the methods were evaluated on the single-cell multi-omics datasets (RFig. 3, i.e., Fig. 4 in current manuscript).*

***RFig. 3 Workflow of the evaluation on single-cell multi-omics datasets.*** ***a*** *Two kinds of omics data were used as input.* ***b*** *Supervised DL methods are evaluated in the classification tasks. The performance of these methods was based on 4-fold cross validation and was evaluated by three metrics: accuracy, F1 macro, and F1 weighted score.* ***c*** *Unsupervised DL methods were first applied to fuse the single-cell multi-omics data to obtain the fused two-dimensional embeddings. Then k-means algorithm was used to cluster the multi-omics dimensionality reduction results into three categories. We employed JI, C-index, silhouette score and Davies Bouldin score as the evaluation indexes of clustering.*

*Applying multi-omics data fusion methods to single-cell multi-omics data provides opportunities to systematically explore the heterogeneity of cells [15]. To further benchmark the performances of DL-based multi-omics data fusion methods, evaluating these methods on single-cell multi-omics data is crucial.*

*The single-cell datasets consisted of two omics data types, i.e., single-cell chromatin accessibility data and single-cell gene expression data. The number of features for these two omics are 49073 and 207203, respectively. And the two omics data were obtained from three different cancer cell lines (HTC, Hela, and K562) for a total of 206 cells [16].*

*Similar to the evaluation on simulated multi-omics data above, firstly, we evaluated six supervised classification methods on the single-cell dataset. The performance of these methods was based on 4-fold cross validation and was evaluated by three metrics: accuracy, F1 macro, and F1 weighted score.*

*For the clustering tasks, ten unsupervised DL methods were first applied to fuse the single-cell multi-omics data to obtain the fused low-dimensional embeddings. Then k-means algorithm was used to cluster the multi-omics dimensionality reduction results into three categories. The clustering of samples is finally obtained to compare the performances of the 10 unsupervised methods. We employed JI, C-index, silhouette score and Davies Bouldin score as the evaluation indexes of clustering.*

Comment 7:

Similar to #5, the final conclusion was not clear for the single-cell data benchmarking. The authors claimed "Overall, lfAE, lfDAE, and efVAE obtained the most promising clustering results among the seven DL methods.", which couldn't be supported by any figure.

*Response：*

*Thank you for the comment. As you mentioned, the conclusion based on different metrics could be inaccurate and unclear. Similar to the response for Comment 5, we defined “unified score” to unify the results of each indicator as the final comprehensive evaluation indicator.*

*According to the benchmarking results based on the “unified score”, it can be seen that moGAT ranked first on the single-cell dataset for the classification tasks, lfAE, lfDAE and efmmdVAE were the top three methods on single-cell datasets for the clustering tasks. (RFig.2, i.e., Fig. 9 in current manuscript)*

Comment 8:

For the TCGA dataset benchmarking, how many samples were included for BRCA and GBM?

*Response：*

*Thank you for the comment. There are 59 and 272 samples were included for BRCA and GBM, respectively.*

*To clearly present the information of each dataset, we have added two tables to summary the datasets (RTable 2, i.e., Table 4 in current manuscript, and RTable 3).*

*In addition, inspired by your comment, we explored the influence of cancer type and data size on model performance more comprehensively.*

*In cancer datasets, we added three additional cancer datasets with different data size and subtyping labels to our classification task datasets. At last, we have five different cancer data sets with ground-truth cancer subtypes from TCGA: breast cancer (BRCA), glioblastoma (GBM), sarcoma (SARC), lung adenocarcinoma (LUAD) and stomach cancer (STAD). Notably, other cancer datasets in TCGA have no ground-truth cancer subtypes labels, so we did not include them in the benchmark of classification tasks. We further investigated how the cancer type and data size influence the benchmarking results. And We have added the corresponding parts in the fourth and fifth paragraphs in “Evaluation of deep learning-based multi-omics data fusion methods on cancer datasets” section of Results in revised manuscript.*

(1) ***The exploration of how cancer type affects performance.***

*Based on the new benchmarking results, it can be seen that all methods do not perform well on BRCA. This is because BRCA has significantly fewer samples than other cancers. For GBM, although it has the largest number of samples among the five cancers, most methods do not achieve good performance. According to investigation, we found that the subtype labels of GBM may have some deviations. Recent researches suggested that GBM should be classified into three subtypes instead of the four subtypes labeled by TCGA [17-20].*

(2) ***The exploration of how data size affects performance.***

*In order to further explore how the data size influence the benchmarks, we reduced the amount of data and observed the effects of data reduction. Specifically, we randomly selected 20%, 40%, 60%, 80% of the total samples in the original data. Then we evaluated all six methods under different amounts of data. The results of the data reduction experiment are shown in RFig. 4 (i.e., Figure S1 in Additional File 1). Except for two GNN-based methods (moGAT and moGCN), the performances of other methods are impaired when the amount of data decreases. The performance of the two GNN-based methods fluctuated greatly. This may be because that the network structure changes greatly with the change of data for the GNN-based methods.*

***RTable 2.*** *The sizes and features of cancer benchmark datasets used in classification task.*

| *Cancers* | *Categories (cancer subtypes)* | *# of samples* | *# of features* |
| --- | --- | --- | --- |
|  |  |  | *Exp, Meth, miRNA* |
| *BRCA* | *Luminal A: 28, Luminal B: 15, Basal-like: 12, HER2-enriched: 4* | *59* | *6000, 5000, 892* |
| *GBM* | *Proneural: 71, Classical: 70, Mesenchymal: 84, Neural: 47* | *272* | *6000, 5000, 534* |
| *SARC* | *DDLPS: 50, LMS: 80, UPS: 44, MFS: 17, MPNST: 5, SS: 10* | *206* | *6000, 5000, 1046* |
| *LUAD* | *TRU: 51, PI: 52, PP: 41* | *144* | *6000, 5000, 554* |
| *STAD* | *EBV: 20, MSI: 38, GS: 43, CIN: 97* | *198* | *6000, 5000, 519* |

*#, number; Exp, gene expression; Meth, DNA methylation; miRNA, miRNA expression; DDLPS, dedifferentiated liposarcoma; LMS, leiomyosarcoma; UPS, undifferentiated pleomorphic sarcoma; MFS, myxofibrosarcoma; MPNST, malignant peripheral nerve sheath tumor; SS, synovial sarcoma; TRU, formerly bronchioid; PI, formerly squamoid; PP, formerly magnoid; EBV, Epstein–Barr virus; MSI, microsatellite instability; GS, genomically stable; CIN, chromosomal instability.*

***RTable 3.*** *The sizes and features of cancer benchmark datasets used in clustering task.*

| *Cancers* | *# of features* | *# of samples* |
| --- | --- | --- |
|  | *Exp, Meth, miRNA* |  |
| *AML* | *6000, 5000, 705* | *169* |
| *BRCA* | *6000, 5000, 1046* | *684* |
| *COAD* | *6000, 5000, 705* | *220* |
| *GBM* | *6000, 5000, 534* | *273* |
| *KIRC* | *6000, 5000, 1046* | *207* |
| *LIHC* | *6000, 5000, 1046* | *409* |
| *LUSC* | *6000, 5000, 1046* | *343* |
| *SKCM* | *6000, 5000, 1046* | *449* |
| *OV* | *6000, 5000, 705* | *290* |
| *SARC* | *6000, 5000, 1046* | *260* |

***RFig. 1 Data reduction experiment on cancer benchmark datasets used in classification task.*** *Accuracy (****a****), F1 macro (****b****), F1 weighted (****c****) of the six unsupervised methods for classification under 20%, 40%, 60%, 80% of the total samples in the original data, respectively.*

*Comment 9:*

*For the clustering benchmarking using TCGA data, were the methods running with 2-6 clusters for each cancer dataset independently? If so, what label was used to calculate the Silhouette and Davies Bouldin score within each cancer type?*

*Response：*

*We are sorry for our obscure expression in the manuscript again. We have re-described this experiment in the current manuscript. To facilitate understanding, we have added a schematic diagram showing how the methods were evaluated on the cancer multi-omics datasets (RFig. 5 and* *RFig. 6, i.e., Fig. 6 and Fig. 8 in current manuscript, respectively).*

***RFig. 5 Workflow of the evaluation on cancer multi-omics datasets.*** ***a*** *Three kinds of omics data were used as input.* ***b*** *Supervised DL methods are evaluated in the classification tasks. The performance of these methods was based on 4-fold cross validation and was evaluated by three metrics: accuracy, F1 macro, and F1 weighted score.* ***c*** *Unsupervised DL methods were first applied to fuse the cancer multi-omics data to obtain the fused 10-dimensional embeddings. Then k-means algorithm was used to cluster the multi-omics dimensionality reduction results into several categories. We employed C-index, silhouette score and Davies Bouldin score as the evaluation indexes of clustering. Furthermore, the associations of the embeddings with survival and clinical annotations were evaluated.*

*We evaluated DL-based multi-omics fusion methods on The Cancer Genome Atlas (TCGA) cancer multi-omics datasets. The multi-omics cancer datasets consist of three omics data types: gene expression, DNA methylation, and miRNA expression.*

*For the classification tasks, we collected five different cancer data sets with ground-truth cancer subtypes from TCGA: breast cancer (BRCA), glioblastoma (GBM), sarcoma (SARC), lung adenocarcinoma (LUAD) and stomach cancer (STAD).*

*For the clustering task, to ensure the authenticity of the assessment, the data we used are from benchmark cancer datasets (http://acgt.cs.tau.ac.il/multi_omic_benchmark/download.html)[21].*

*Similar to the evaluation on simulated multi-omics data and single-cell multi-omics data above, we firstly evaluated six supervised classification methods on the five different cancer data sets with ground-truth cancer subtypes. The performance of these methods was based on 4-fold cross validation and was evaluated by three metrics: accuracy, F1 macro, and F1 weighted score (see Methods).*

*For the clustering tasks, ten unsupervised DL methods were first applied to fuse the cancer multi-omics data to obtain the fused 10-dimensional embeddings. The embedding dimension is set as in the work of Bismeijer et al.[22] and Cantini et al. [23]. Then k-means algorithm was used to cluster the multi-omics dimensionality reduction results into several categories. Because the optimal cluster number (the ground-truth cancer subtypes) is uncertain, the number of clusters is set from two to six in this study. The clustering of the samples is finally obtained to compare the performances of the 10 unsupervised methods. We employed C-index, silhouette score and Davies Bouldin score as the evaluation indexes of clustering. Notably, JI was not used as the evaluation index because of the lack of the information of ground-truth cancer subtypes in the benchmark cancer datasets.*

*To further evaluate the effect of data fusion by using these DL-based methods, we not only used the fused ten-dimensional embeddings for clustering analysis, but also evaluated the associations of the embeddings with survival and clinical annotations.*

Comment 10:

The analysis and interpretation of Fig4c and 4d might be inappropriate. The authors first performed dimensionality reduction to reduce the features into ten and then associated these ten features with clinical outcomes and annotations. However, the number of significant associations from the ten features did not reflect any kind of performance. The ten features in the latent space are likely to be orthogonal. Having all ten features significantly associated with any one specific clinical feature means all ten features captured similar information, which may indicate an inferior performance of the dimensionality reduction.

*Response：*

*Thank you for the important comment. We are sorry that there isn’t a clear description about these two evaluations in the current manuscript. To avoid* *ambiguity, we re-described the corresponding part in current manuscript. Below we give detailed explains on these two analyses in the benchmark (RFig. 6, i.e., Fig. 8 in current manuscript).*

***RFig. 6 Graphic summary of the cancer sub-benchmark.*** ***a*** *The details of testing the association of embeddings with survival.* ***b*** *The details of testing the association of embeddings with clinical annotations.*

*To further* *assess the effect of data fusion by using these DL-based methods, we evaluated the associations of the embeddings with survival and clinical annotations. On the one hand, the associations could reflect representational ability of the fused ten-dimensional embeddings, and on the other hand, the associations partly reflect the interpretability of the embeddings.*

*First, to evaluate the association of the embeddings with survival, we employed the Cox proportional-hazards regression model and calculated the Bonferroni-corrected p-values. The Bonferroni-corrected p-values indicates to what extent an embedding can distinguish the difference between the population survival conditions. The more the embeddings with strong association with survival (the Bonferroni-corrected p-values smaller than 0.05), the better the performance of the method. In fact, survival is a comprehensive clinical characteristic affected by many factors. For example, the survival markers of poor prognosis for adrenocortical cancer can be various kinds of gene, miRNA and DNA methylation signatures [24-27]. These survival markers are actually not similar to each other, and indicate the survival prognosis of cancer from different aspects. Similar to the survival markers, the embeddings with strong association with survival can reflect their impact on survival from various aspects.*

*However, in order to avoid the similarity between latent embeddings as you mentioned, the next analysis defined selectivity score to find the latent embeddings with sufficient differentiation and avoid the similarity between them.*

*Using the same ten-dimensional embeddings described above, we further evaluated the association of the embeddings with clinical annotations. We used the Kruskal-Wallis test and Wilcoxon rank-sum to test the significance of the associations of the embeddings with several clinical annotations. Different from the association of the embeddings with survival mentioned above, the association of the embeddings with clinical annotations is expected to be one-to-one mapping, that is, an embedding is associated with a clinical annotation. In this way, each embedding can be interpretable. Therefore, after obtaining the strong associations by Kruskal-Wallis test and Wilcoxon rank-sum, we employed the selectivity score as the evaluation metric [23]. The selectivity score is in the range of [0,1]. When each embedding is associated with one and only one clinical annotation, the selectivity score is 1. When all features are associated with all clinical annotations, the selectivity score is 0. The evaluation based on selectivity score could reflect whether the latent embeddings have sufficient differentiation.*

*Given all that, we think these two evaluations could be used as a supplement to clustering performance evaluation.*

Comment 11:

Fig5 should quantitatively summarise the evaluations, not qualitatively as in the current form.

*Response：*

*Thanks for this detailed and important advice.*

*As you suggested, to quantitatively summarize the benchmarking results, we used “unified score” to unify the results of each indicator as the final quantitatively comprehensive evaluation indicator (RFig. 2, i.e., Fig. 9 in current manuscript). The unified score is referring to the rank aggregation scheme for the Synapse Challenge (https://doi.org/10.7303/syn6131484). If one method was evaluated in more than one scenario (e.g., in simulated multi-omics dataset, all methods are evaluated on six scenarios: three cluster number × same size/random size), we used its average unified scores. The definition of “unified score” was added in the section “Evaluation Metrics” in Methods in current manuscript. We have added the benchmarking results based on the “unified score” in the third paragraph in “Discussion and Conclusion” section in current manuscript. And we modified old version Fig. 5 in the current manuscript (i.e., Fig. 9 in current manuscript).*

Comment 12:

For each of the three experiments, how was the evaluation conducted? How much data were used in the test set? Was cross-validation used?

*Response：*

*Thank you for the constructive suggestion. Due to our negligence, the contents were not described clearly in the current manuscript. For each of the three experiments, we hope that we have answered all specific concerns in response of Comments 3&6&9. Meanwhile, we have added the experiment details in the corresponding parts. We also have added tables and figures for ease of understanding (RTable 2, RFig. 1, RFig. 3, RFig. 5 and RFig. 6, i.e., Table 4, Fig. 2, Fig. 4, Fig. 6 and Fig. 8 in current manuscript).*

*(1) On simulated datasets, we evaluated the methods through two tasks: classification task and clustering task. First, supervised DL methods are evaluated in the classification tasks. The performance of these methods was based on 4-fold cross validation and was evaluated by three metrics: accuracy, F1 macro, and F1 weighted score. Then, unsupervised DL methods are applied to fuse the simulated multi-omics data to obtain low-dimensional embeddings first. After that, k-means algorithm is used to cluster the multi-omics dimensionality reduction results. We employed Jaccard Index, C-index, silhouette score and Davies Bouldin score as the evaluation indexes of clustering.*

*(2) The evaluation on single-cell datasets used the same methodology as on simulated datasets (RFig. 3, i.e., Fig. 4 in current manuscript).*

*(3) On cancer datasets, first, supervised DL methods are evaluated in the classification tasks. The performance of these methods was based on 4-fold cross validation and was evaluated by three metrics: accuracy, F1 macro, and F1 weighted score. Then unsupervised DL methods were first applied to fuse the cancer multi-omics data to obtain the fused low-dimensional embeddings. After that, k-means algorithm was used to cluster the multi-omics dimensionality reduction results into several categories. We employed C-index, silhouette score and Davies Bouldin score as the evaluation indexes of clustering. Furthermore, the associations of the embeddings with survival and clinical annotations were evaluated. To evaluate the association of the embeddings with survival, we employed the Cox proportional-hazards regression model and calculated the Bonferroni-corrected p-values. To evaluate the association of the embeddings with clinical annotations, we used the Kruskal-Wallis test and Wilcoxon rank-sum to test the significance of the associations of the embeddings with these clinical annotations and defined the selectivity score as the evaluation metric.*

**Minor points:**

Comment 1:

In Table 1, row 1 and 3 are identical?

*Response：*

*Thank you for reading so carefully. We are very sorry for making this confusion. We have revised this table (RTable 8, i.e., Table 1 in current manuscript). And the table has been modified in the current manuscript.*

***RTable 8.*** *Evaluation of the DL-based multi-omics fusion methods and metrics*

| *Dataset* | | *Evaluation model* | *Task type* | *Evaluation metrics* |
| --- | --- | --- | --- | --- |
| ***Simulated multi-modal datasets*** | | *lfNN, efNN, lfCNN, efCNN, moGCN* | *Classification* | *1. Accuracy*  *2. F1 macro*  *3. F1 weighted* |
|  |  | *lfAE, efAE, lfDAE, efDAE, lfVAE, efVAE, lfSVAE, efSVAE, lfmmdVAE, efmmdVAE* | *Clustering* | *1. Jaccard Index*  *2. C-index*  *3. Silhouette score*  *4. Davies Bouldin score* |
| ***Single-cell multi-modal datasets*** | | *lfNN, efNN, lfCNN, efCNN, moGCN* | *Classification* | *1. Accuracy*  *2. F1 macro*  *3. F1 weighted* |
|  |  | *lfAE, efAE, lfDAE, efDAE, lfVAE, efVAE, lfSVAE, efSVAE, lfmmdVAE, efmmdVAE* | *Clustering* | *1. Jaccard Index*  *2. C-index*  *3. Silhouette Score*  *4. Davies Bouldin Score* |
| ***Cancer multi-modal datasets*** | *BRCA, GBM, SARC, LUAD, STAD* | *lfNN, efNN, lfCNN, efCNN, moGCN* | *Classification* | *1. Accuracy*  *2. F1 macro*  *3. F1 weighted* |
|  | *AML, BRCA, COAD, GBM, KIRC, LIHC, LUSC, SKCM, OV, SARC,* | *lfAE, efAE, lfDAE, efDAE, lfVAE, efVAE, lfSVAE, efSVAE, lfmmdVAE, efmmdVAE* | *Clustering* | *1. C-index*  *2. Silhouette Score*  *3. Davies Bouldin Score* |
|  |  |  | *Association of embeddings with survival* | *Cox proportional-hazards regression* |
|  |  |  | *Association of embeddings with clinical annotations* | *Selectivity score* |

Comment 2:

The authors included computational details for each benchmarked method, but these details can be referred to the original publication. These details might be necessary.

*Response：*

*Thank you for your advice. As you mentioned, more computational details can be found in the literature of the original model. But because the original methods are aimed at different tasks, the description of computational details varies greatly. We extracted the core computational processes from the original methods and adjusted the computational details in Methods part. Meanwhile, the references of original publications have been added in the current manuscript.*

Comment 3:

"To ensure the authenticity of the assessment, the data we used are all from benchmark cancer datasets", does the referenced website has the up-to-date data, as the data are not directly downloaded from TCGA? Also BRCA is not listed on that page. What is the source of the breast cancer data?

*Response：*

*Thank you for this detailed comment. By checking the data from referenced website and TCGA, we found these data are the same as the up-to-date TCGA data.*

*The “breast cancer” we used in original manuscript is actually BRCA. We modified the cancer names to the unified standard form in TCGA in the revised manuscript.*

***Reference***

*1.	Kuru Hb, Tastan O, Cicek E. MatchMaker: a deep learning framework for drug synergy prediction. IEEE ACM T Comput Bi. 2021:1545-5963.*

*2.	Preuer K, Lewis RPI, Hochreiter S, Bender A, Bulusu KC, Klambauer G. DeepSynergy: predicting anti-cancer drug synergy with Deep Learning. Bioinformatics. 2018;34(9):1538-46.*

*3.	Islam MM, Huang S, Ajwad R, Chi C, Wang Y, Hu P. An integrative deep learning framework for classifying molecular subtypes of breast cancer. Comput Struct Biotechnol J. 2020;18:2185-99.*

*4.	Fu Y, Xu J, Tang Z, Wang L, Yin D, Fan Y, et al. A gene prioritization method based on a swine multi-omics knowledgebase and a deep learning model. Commun Biol. 2020;3(1):1-11.*

*5.	Wang T, Shao W, Huang Z, Tang H, Zhang J, Ding Z, et al. MOGONET integrates multi-omics data using graph convolutional networks allowing patient classification and biomarker identification. Nat Commun. 2021;12(1):3445.*

*6.	Xing X, Yang F, Li H, Zhang J, Zhao Y, Gao M, et al.: An Interpretable Multi-Level Enhanced Graph Attention Network for Disease Diagnosis with Gene Expression Data. In: 2021 IEEE International Conference on Bioinformatics and Biomedicine (BIBM). 2021: 556-61.*

*7.	Ma T, Zhang A. Integrate multi-omics data with biological interaction networks using Multi-view Factorization AutoEncoder (MAE). BMC Genomics. 2019;20(S11):944.*

*8.	Lee TY, Huang KY, Chuang CH, Lee, , C. Y., Chang TH. Incorporating deep learning and multi-omics autoencoding for analysis of lung adenocarcinoma prognostication. Comput Biol Chem. 2020;87:107277.*

*9.	Poirion OB, Chaudhary K, Garmire LX. Deep Learning data integration for better risk stratification models of bladder cancer. AMIA Jt Summits Transl Sci Proc. 2018;2018:197-206.*

*10.	Guo L-Y, Wu A-H, Wang Y-x, Zhang L-p, Chai H, Liang X-F. Deep learning-based ovarian cancer subtypes identification using multi-omics data. BioData Min. 2020;13(1):10.*

*11.	Zhang X, Zhang J, Sun K, Yang X, Dai C, Guo Y. Integrated multi-omics analysis using variational autoencoders: application to pan-cancer classification. IEEE Int Conf Bioinformatics Biomed. 2019:765-9.*

*12.	Ronen J, Hayat S, Akalin A. Evaluation of colorectal cancer subtypes and cell lines using deep learning. Life Sci Alliance. 2019;2(6):1-16.*

*13.	Hira MT, Razzaque MA, Angione C, Scrivens J, Sawan S, Sarkar M. Integrated multi-omics analysis of ovarian cancer using variational autoencoders. Sci Rep. 2021;11(1):6265.*

*14.	Chalise P, Raghavan R, Fridley BL. InterSIM: Simulation tool for multiple integrative ‘omic datasets’. Comput Methods Programs Biomed. 2016;128:69-74.*

*15.	Lee J, Hyeon DY, Hwang D. Single-cell multiomics: technologies and data analysis methods. Exp Mol Med. 2020;52(9):1428-42.*

*16.	Liu L, Liu C, Quintero A, Wu L, Yuan Y, Wang M, et al. Deconvolution of single-cell multi-omics layers reveals regulatory heterogeneity. Nat Commun. 2019;10(1):470.*

*17.	Mao XG, Xue XY, Wang L, Lin W, Zhang X. Deep learning identified glioblastoma subtypes based on internal genomic expression ranks. BMC Cancer. 2022;22(1):86.*

*18.	Wang Q, Hu B, Hu X, Kim H, Squatrito M, Scarpace L, et al. Tumor Evolution of Glioma-Intrinsic Gene Expression Subtypes Associates with Immunological Changes in the Microenvironment. Cancer Cell. 2017;32(1):42-56 e6.*

*19.	Hu B, Ruan Y, Wei F, Qin G. Identification of three glioblastoma subtypes and a six-gene prognostic risk index based on the expression of growth factors and cytokines. Am J Transl Res. 2020;12(8):4669-82.*

*20.	Zhang P, Xia Q, Liu L, Li S, Dong L. Current Opinion on Molecular Characterization for GBM Classification in Guiding Clinical Diagnosis, Prognosis, and Therapy. Front Mol Biosci. 2020;7:562798.*

*21.	Rappoport N, Shamir R. Multi-omic and multi-view clustering algorithms: review and cancer benchmark. Nucleic Acids Res. 2018;46(20):10546-62.*

*22.	Bismeijer T, Canisius S, Wessels LFA. Molecular characterization of breast and lung tumors by integration of multiple data types with functional sparse-factor analysis. PLoS Comput Biol. 2018;14(10):e1006520.*

*23.	Cantini L, Zakeri P, Hernandez C, Naldi A, Thieffry D, Remy E, et al. Benchmarking joint multi-omics dimensionality reduction approaches for the study of cancer. 2021;12(1):124.*

*24.	Mizdrak M, Ticinovic Kurir T, Bozic J. The Role of Biomarkers in Adrenocortical Carcinoma: A Review of Current Evidence and Future Perspectives. Biomedicines. 2021;9(2).*

*25.	Jouinot A, Assie G, Libe R, Fassnacht M, Papathomas T, Barreau O, et al. DNA Methylation Is an Independent Prognostic Marker of Survival in Adrenocortical Cancer. J Clin Endocrinol Metab. 2017;102(3):923-32.*

*26.	Cherradi N. microRNAs as Potential Biomarkers in Adrenocortical Cancer: Progress and Challenges. Front Endocrinol (Lausanne). 2015;6:195.*

*27.	Wen Y, Song X, Yan B, Yang X, Wu L, Leng D, et al. Multi-dimensional data integration algorithm based on random walk with restart. BMC Bioinformatics. 2021;22(1):97.*

**Second round of review**

**Reviewer 1**

Basically, I feel that the authors have effectively addressed the criticisms raised in the initial review. I recommend publication.

**Reviewer 2**

The authors have addressed most of my comments.

However, the naming of the methods is still debatable.

Also, it is still not clear what is to be classified for the supervised task.
